# Hyper-Cross-Linked Microporous Polymers as Cheap and Efficient Catalysts for the Synthesis of Biodiesel

**DOI:** 10.1021/acsaenm.6c00096

**Published:** 2026-03-25

**Authors:** C. Grazia Bezzu, Natasha Hawkins, Rebecca Foster, Ariana R. Antonangelo, James W. Ryan, Anna Williamson, Mariolino Carta

**Affiliations:** † Department of Chemistry, Faculty of Science and Engineering, 16379 Swansea University, Singleton Park, Swansea SA28PP, U.K.; ‡ Instituto de Síntesis Química y Catálisis Homogènea, Facultad de Ciencias, CSIC-Universidad de Zaragoza, C/Pedro Cerbuna 12, Zaragoza 50009, Spain

**Keywords:** Hyper-cross-linked polymers, Microporous materials, Transesterification, Biodiesel, Catalysis

## Abstract

In this work, we report a series of hyper-cross-linked sulfonated polymers, structurally related to PIMs, as highly efficient and versatile catalysts for biodiesel production. By tuning monomer composition to increase aromatic content, we generate porous polymers with tailored surface areas and porosity, which are thoroughly characterized and assessed for CO_2_/N_2_ separation. These catalysts promote both Fischer esterification of free fatty acids and transesterification of triglyceride oils derived from the same fatty acids, achieving over 95% conversion to fatty acid methyl esters (FAME) in 24 h or less under diverse conditions. Remarkably, by using a variety of oils, high activity is maintained even with reduced catalyst and methanol loadings, demonstrating intrinsic efficiency and robustness. The polymers prove to be fully scalable and recyclable, retaining performance over multiple cycles and efficiently converting waste cooking sunflower oil with comparable yields to pure edible oils. This work establishes a direct structure–property–performance relationship, linking polymer architecture and porosity to catalytic activity, and provides a versatile, sustainable platform for next-generation porous catalysts in biodiesel production and broader chemical transformations.

## Graphical abstract

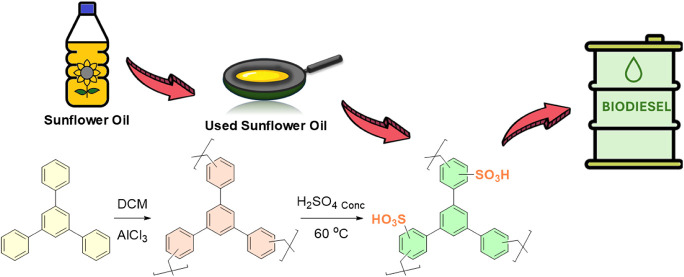

## Introduction

In response to increasing awareness of the global warming crisis, the Climate Change Committee has committed to achieve net-zero emissions by 2050,^1,2^ target widely viewed as essential for ensuring long-term environmental sustainability. A multifaceted approach involving international agreements, governmental policies, technological innovation, and individual actions, has been implemented. Despite the ongoing large-scale transition from fossil fuels to clean energy sources, and the adoption of energy-efficient strategies including enhanced building insulation, more efficient appliances, smart grids and sustainable transport, significant research and development is still needed to improve renewable-energy production and storage. In addition, a global retrofitting program would be both complex and costly.^3,4^ This is because the vast majority of renewable energies have an electricity output instead of a physical fuel (coal, petrol, gas, etc.), and so all the technologies that rely on physical fuels are expected to become obsolete, or new alternatives will be needed.^5,6^ Retrofitting is likely to be a long and complex problem in the net zero target, therefore a temporary solution is needed.^7^ Biofuels, which include bioethanol, biodiesel, biogas, biomass, etc. are combustible material that are derived from biological matter, mainly plants, that can be directly employed in current technologies. Biodiesels are a subgroup of biofuels that are predominantly made of fatty acid methyl esters (FAMEs).^8^ As the name suggests, they are similar to fossil fuel derived diesel, and can be used in existing diesel engines. They are synthesized from long chained fatty acids (lipids) that are typically derived from plant oils via transesterification reactions, and the process can be facilitated by either an acid or base catalyst. In industrial applications, bases such as sodium hydroxide and potassium hydroxide, and acids such as sulfuric, are used as homogeneous base catalysts. This is undesirable not only because of the drawbacks associated with homogeneous catalysis (i.e., expensive separation of products from catalysts, unreacted materials, and byproducts), but also because the strong bases and acids are harsh (corrosive) and unsustainable.^9^ Heterogeneous catalysts, instead, work in mild conditions and are easily removed and recycled from the reaction by simple filtration. Research into heterogeneous catalysts for biodiesel synthesis is dominated by acid catalysts,^10^ but also basic polymers prove to be competitive.^11–13^ Typically, a good heterogeneous catalyst is supported onto a porous material, so that the conversions rate increases because the substrates can be confined into the pores and react more quickly. However, these insoluble catalysts are often prepared by simply anchoring the active sites onto a polymeric support, which may cause leaching of the material into the solution and makes its recycling and reusing more problematic and costly.^14^ Because of the ease of their synthesis, the high BET surface areas and internal free volume (IFV), networked insoluble hyper-cross-linked PIM-like polymers offer important advantages over standard polymeric supports used for catalysis, providing an attractive solution to conventional materials.^15^ In these polymers the porosity is typically induced by a combination of their inefficient packing in the solid state, and their hyper-cross-linked nature. They contribute in leaving voids of nanodimensions that can be used for a wide range of applications, including gas separation and storage,^16^ water purification,^17^ and catalysis.^18,19^ We anticipate significant advantages in employing insoluble polymers functionalized with acid substituents for the efficient synthesis of biodiesel. Hyper-cross-linked polymers (HCPs) have been trialedas catalysts for biodiesel. For example, Bhunia et al. report the synthesis of HMP-1-SO_3_H, an acid catalyst, prepared from carbazole and x,x′-dibromo-*p*-xylene. This material exhibited a high yield of biodiesel products under mild reaction conditions at room temperature.^20^ Dawson and coworkers reported a series of conjugated microporous polymers (CMPs) based on the sulfonation of bromophenol blue (BB) and bromocresol green (BG), which were successfully used for both the esterification of fatty acids and the transesterification of various oils.^21^ Kalla et al. reported the synthesis of pBPA-SO_3_H, an HCP prepared from bisphenol A monomers reaching conversions between 94 and 97% using palmitic, stearic, and oleic acids respectively at 25 °C.^22^ Recently, we reported the synthesis and characterization of a series of functionalized hyper-cross-linked PIMs, which were used for gas separation.^17,23^ Given the well-established catalytic activity of acidic functionalities in biodiesel production, this study explores a series of sulfonated hyper-cross-linked polymers (HCPs) as effective heterogeneous acid catalysts for biodiesel synthesis. These materials were evaluated in both the esterification of free fatty acids and the transesterification of various commercially available oils. Crucially, we also conducted catalytic tests using recycled (used) cooking oils, an essential step toward promoting sustainable biodiesel production by avoiding the use of edible feedstock.

## Experimental Part

### Synthesis of TPB-HC, HPB-HC, TPB-SO_3_H, and HPB-SO_3_H

The synthesis and functionalization of hyper-cross-linked polymers, apart from the novel *m*-TER were reproduced according to our previously reported procedure but scaled up.^1,2^

#### HPB-HC^3^

HPB (10.0 g, 18.7 mmol) and AlCl_3_ (24.9 g, 187 mmol) were added to DCM (500 mL) and stirred at reflux for 24 h under a nitrogen atmosphere. The solution was filtered, and the obtained powder washed with plenty of water and ethanol. The powder was washed sequentially via reflux with ethanol, chloroform, THF, acetone, and methanol, and finally dried in a vacuum oven at 100 °C for 20 h. The HPB-network polymer (13.0 g, 21.2 mmol, ∼100% was analyzed by IR spectroscopy, TGA, and BET. SA_BET_ = 1932 m^2^g^–1^, TGA: Thermal degradation commences at 295 °C. FTIR-ATR (cm^–1^): 1096, 1452, 2978. ^13^C MAS SSNMR (101 MHz) δ (ppm) 136.69, 131.03, 126.72, 82.72, 72.49, 65.76, 54.44, 40.27, 34.15, 13.10.

#### HPB-SO_3_H^1^

HPB-HC (5.0 g, 8.16 mmol) was added to H_2_SO_4_ (100 mL), stirred for 30 min, and heated to 60 °C for 8 h under a nitrogen atmosphere. The solution was cooled to room temperature, poured over ice, and filtered. The solid was washed via heating to 60 °C in deionized water twice and filtered until the filtrate showed a neutral pH. The solid was refluxed in methanol twice, filtered, and dried in a vacuum oven at 60 °C for 18 h. The obtained dark powder was analyzed (5.58 g, 8.16 mmol, ∼100%; the yield is considered for a single sulfonic group, but the titration shows 1.16 per repeat unit). SA_BET_ = 1404 m^2^g^–1^, TGA: Thermal degradation commences at 165 °C. FTIR-ATR (cm^–1^): 1039, 1175, 2980, 3420. ^13^C MAS SSNMR (101 MHz) δ (ppm) 255.64, 235.97, 192.27, 135.92, 131.84, 72.44, 55.35, 34.28, 14.61.

#### TPB-HC^2^

TPB (10.0 g, 32.6 mmol) and AlCl_3_ (43.5 g, 326 mmol) were added to DCM (500 mL) and stirred at reflux for 24 h under a nitrogen atmosphere. The solution was filtered, and the obtained powder washed with plenty of water and ethanol. The powder was washed sequentially via reflux with ethanol, chloroform, THF, acetone, and methanol, and finally dried in a vacuum oven at 100 °C for 20 h. (11.3 g, 32.6 mmol, ∼100%. BET = 2540 m^2^ g^–1^; FT-IR ν max (cm^–1^) 2968, 1700, 1600, 1392, 1048, 872. ^13^C MAS SSNMR (101 MHz) δ (ppm) 162.5, 139.4, 131.6, 56.9, 35.3, 14.7, −12.9.

#### TPB-SO_3_H^2^

TPB-HC (5.0 g, 14.5 mmol) was added to H_2_SO_4_ (100 mL), stirred for 30 min, and heated to 60 °C for 8 h under a nitrogen atmosphere. The solution was cooled to room temperature, poured over ice, and filtered. The solid was washed via heating to 60 °C in deionized water twice and filtered until the filtrate showed a neutral pH. The solid was refluxed in methanol twice, filtered, and dried in a vacuum oven at 60 °C for 18 h. The obtained dark powder was analyzed (5.3 g, 12.45 mmol, ∼85%. BET = 1585 m^2^ g^–1^, total pore volume = 0.8521 (at P/P_0_ = 0.9774); CO_2_ adsorption at 273 K/1 bar = 298 mg g^–1^ (6.8 mmol g^–1^); TGA: initial mass loss at 180 °C. FT-IR ν max (cm^–1^) 2982, 1700, 1589, 1240, 1238, 1165, 1033, 606. ^13^C MAS SSNMR (101 MHz) δ (ppm) 189.7, 138.0, 132.4, 79.6, 72.4, 69.5, 54.5, 48.5, 35.8, 15.2.

#### *M*-TER

*m*-TER monomer (10.0 g, 43.4 mmol) and AlCl_3_ (57.9 g, 434 mmol) were added to DCM (500 mL). The mixture was refluxed for 24 h under a nitrogen atmosphere. The solution was quenched into water, filtered, and washed with water. The solid swelled in ammonia and filtered. Then refluxed in ammonia for 20 min, filtered and washed with water. The obtained powder was then washed consecutively via reflux with ethanol, chloroform, THF, acetone and methanol, twice each with swelling of each solvent between each reflux. The brown-red powder was dried at 120 °C for 18 h (13.2 g, 49.0 mmol, ∼100%. IR (cm^–1^): 3014, 2884, 1700, 1600, 1435, 1403. TGA: Thermal degradation commences at: 303 °C. SA_BET_: 1977m^2^g^–1^; Total pore volume = 1.190 (at P/P_0_ ∼ 0.98); ^13^C MAS SSNMR (101 MHz) δ (ppm) 141.03, 132.05, 127.48, 58.40, 35.23, 16.30.

#### *m*-TER-SO_3_H

*m*-TER (5.0 g, 18.6 mmol) was added to H_2_SO_4_ (100 mL), stirred for 30 min, and heated to 60 °C for 8 h under a nitrogen atmosphere. The solution was cooled to room temperature, poured over ice, and filtered. The solid was washed via heating to 60 °C in deionized water twice and filtered until the filtrate showed a neutral pH. The solid was refluxed in methanol twice, filtered, and dried in a vacuum oven at 60 °C for 18 h. The obtained dark blue-black powder was analyzed (5.9 g, 16.7 mmol, 90%. IR (cm^–1^): 3325, 2928, 1675, 1593, 1168, 1032, 892, 613. TGA: Thermal degradation commences at: 177 °C SA_BET_: 1100 m^2^g^–1^; Total pore volume = 0.4975 (at P/P_0_ ∼ 0.98). ^13^C MAS SSNMR (101 MHz) δ (ppm) 188.23, 139.09, 132.60, 53.92, 36.93, 18.25.

#### Catalysis Tests

Reactions, for both esterifications and transesterifications, were carried out in glass tubes equipped with magnetic stirrer bars and fitted with water-cooled reflux condensers. The vessels were heated at 60 °C using a four-position temperature-controlled metal heating block filled with sand, allowing parallel reactions to be conducted under identical conditions with continuous stirring.

Esterification of Acids. One mmol of acid, methanol (2 mL) and catalyst (10 mg) were stirred together at 60 °C. Samples (20 μL) were taken at regular intervals. Prior to measurement, excess methanol was removed from the sampled amount under a gentle stream of nitrogen, and the conversion was monitored via NMR (CDCl_3_).

Transesterification of oils. 100 mg of each oil, methanol (5.7 mL) and catalyst (60 mg) were stirred at 60 °C. Samples (20 μL) were taken at regular intervals. Prior to measurement, excess methanol was removed from the sampled amount under a gentle stream of nitrogen, and the conversion was monitored via NMR (CDCl_3_).

## Results and Discussion

### Synthesis

The polymers were prepared according to our previously published synthesis.^23^ The hydrocarbon monomers were selected based on a specific structural design strategy involving an increasing number of aromatic rings. The chosen monomers include: ***m*** **-terphenyl (** ***m*** **-Ter)**, composed of three aromatic rings arranged in a bent linear configuration; **1,3,5-tris­(4-phenyl)­benzene (TPB)**, consisting of four aromatic rings forming a trigonal geometry; and **hexaphenylbenzene (HPB)**, containing six peripheral phenyl rings attached to a central benzene core, resulting in a paddlewheel-like structure. These commercial monomers were polymerized in the presence of dichloromethane (DCM), which acts as both the solvent and the cross-linker via a Friedel–Crafts reaction and AlCl_3_ as the Lewis acid (Figure 1).^23^ To achieve sulfonation the hydrocarbon polymer was reacted with concentrated sulfuric acid at 60 °C. Titration indicated roughly 0.8–1.16 sulfonic groups per repeated unit, with **HPB** displaying a higher amount of acidic moieties followed by respectively **TPB** and ***m*** **-Ter**. The structural properties of all polymers were characterized and are reported in the Supporting Information. Polymers based on HPB and TPB, including both hydrocarbon and sulfonated versions, were previously described in detail;^23^ for completeness and direct comparison, all spectra are reproduced in this study. All polymers were found to be completely insoluble, as expected, and were therefore characterized using FT-IR and ^13^C MAS SSNMR (Figures S38–S40 and S26–S31, respectively). FT-IR spectra show a marked increase in aliphatic C–H stretching vibrations around 3000 cm^–1^, indicating the efficient formation of cross-links via the Friedel–Crafts reaction. This is consistent with prior reports on hyper-cross-linked polymers of this type.^24,25^ The introduction of sulfonic acid groups is confirmed by characteristic S=O stretching vibrations between 1150 and 1030 cm^–1^. Analysis of the ^13^C MAS SSNMR spectra further support the polymer structure and functionalization. Broad resonances between 160 and 130 ppm correspond to aromatic carbons, whereas several smaller peaks between 75 and 15 ppm are attributed to methylene cross-linkers. Some of the aromatic resonances are slightly shifted, likely due to the proximity of polar functional groups. Although SSNMR cannot directly detect sulfonic groups, the observed minor shift of the aromatic resonances near 135 ppm supports modifications of the aromatic environment consistent with sulfonation. The presence of sulfonic acid groups is thus confirmed through FT-IR analysis and further validated by acid–base titration. Thermogravimetric analysis (TGA, Figures S1 and S2) shows the typical thermal profile of hyper-cross-linked polymers, as previously observed.^23^ The hydrocarbon polymers begin to degrade around 290–300 °C, whereas the sulfonated versions exhibit an initial weight loss between 160 and 180 °C, corresponding to the decomposition of sulfonic acid groups. All polymers leave a char residue of approximately 75–80%, demonstrating their high thermal stability.

**Figure 1:**
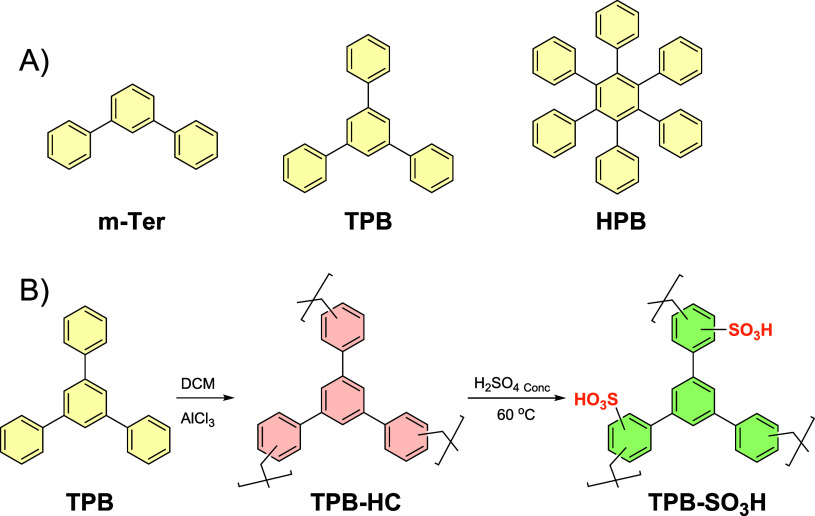
(**A**) Core units of the functionalized polymers; (**B**) example of polymerization and functionalization.

### Textural Properties

The main adsorption properties of the polymers were assessed via the typical isothermal adsorption of N_2_ at 77 K, to obtain the BET surface area, and CO_2_ at 273 K, to evaluate the pore size distribution. The porosity properties were thoroughly assessed and we found that for **TPB** and **HPB** polymers they are in line with the results obtained in our previous paper (Table 1).^23^ The brand new ***m*** **-Ter** polymer results are herein reported for the first time, and summarized in Table 1.

**Table 1:** Textural Properties of Polymers Used for This Work

	N_2_ 77 K		CO_2_ Adsorption		IAST Selectivity	Q_st_
Polymer	SA_BET_ (m^2^g^–1^)	Pore Volume (cm^3^g^–1^)	273 K (mg g^–1^)	298 K (mg g^–1^)	CO_2_/N_2_	(KJ/mmol)
HPB-HC	1933	1.630	196	111	13.5	21
HPB-SO_3_H	1404	1.310	175	112	18.7	28
TPB-HC	2540	1.300	220	121	14.1	25
TPB-SO_3_H	1585	0.852	298	179	17.9	29
*m*-TER-HC	1977	1.190	219	126	14.5	31
*m*-TER-SO_3_H	1100	0.497	200	125	27.8	33

For the purpose of this study, and to assess how much the porosity helps the catalysis performance, we employed monomers which typically produced high performing microporous materials in earlier works.^26–28^ The structures of the three monomers were intentionally designed to be similar, with an increasing aromatic content in each one. This was expected to influence the degree of sulfonation following the initial Friedel–Crafts polymerization, which yielded the hydrocarbon-based polymers. The purely hydrocarbon hyper-cross-linked polymers, as already reported and herein confirmed, provided much higher BET surface areas compared to the sulfonated ones. Of particular importance is the synthesis of the novel ***m*** **-Ter** (Figure 1A), which was specifically selected due to its low cost and ready availability as a starting material for the preparation of high-performance microporous polymers. Looking closely at the textural properties, it is plausible that the acidic functional groups, apart from filling the pores, tend to reduce the final surface areas also by engaging in strong intramolecular hydrogen bonding.^29–31^ The BET surface areas of the newly synthesized polymers were found to be comparable to those of previously reported HCP-PIMs, with only minor differences, primarily observed in the CO_2_ adsorption uptake of the HPB-based polymers. Single isotherms showing N_2_ adsorption at 77 K and CO_2_ adsorptions at 273 and 298 K are reported in Figures S3–S14. The ***m*** **-Ter**-based polymer exhibited a similar trend, displaying a high BET surface area in its hydrocarbon form consistent with the other materials, followed by a reduction in porosity upon sulfonation (a 45% reduction in BET surface area). Notably, the use of ***m*** **-Ter**, which is an extremely inexpensive and readily available monomer, resulted in performance that was surprisingly competitive compared to more costly counterparts. This outcome was somewhat unexpected, as the increased rotational freedom around the aromatic moieties in ***m*** **-Ter** was anticipated to lower both the surface area and CO_2_ uptake. However, as shown in Table 1 and Figure 2, the material demonstrated excellent CO_2_ adsorption (**A**) capacities of approximately 200 mg g^–1^ (4.55 mmol g^–1^) at 273 K and 125 mg g^–1^ (2.85 mmol g^–1^) at 298 K. Given the strong affinity of sulfonic acid groups for CO_2_,^23^ we also evaluated the CO_2_/N_2_ selectivity of the sulfonated ***m*** **-Ter** polymer using Ideal Adsorbed Solution Theory (IAST, **B**).

**Figure 2:**
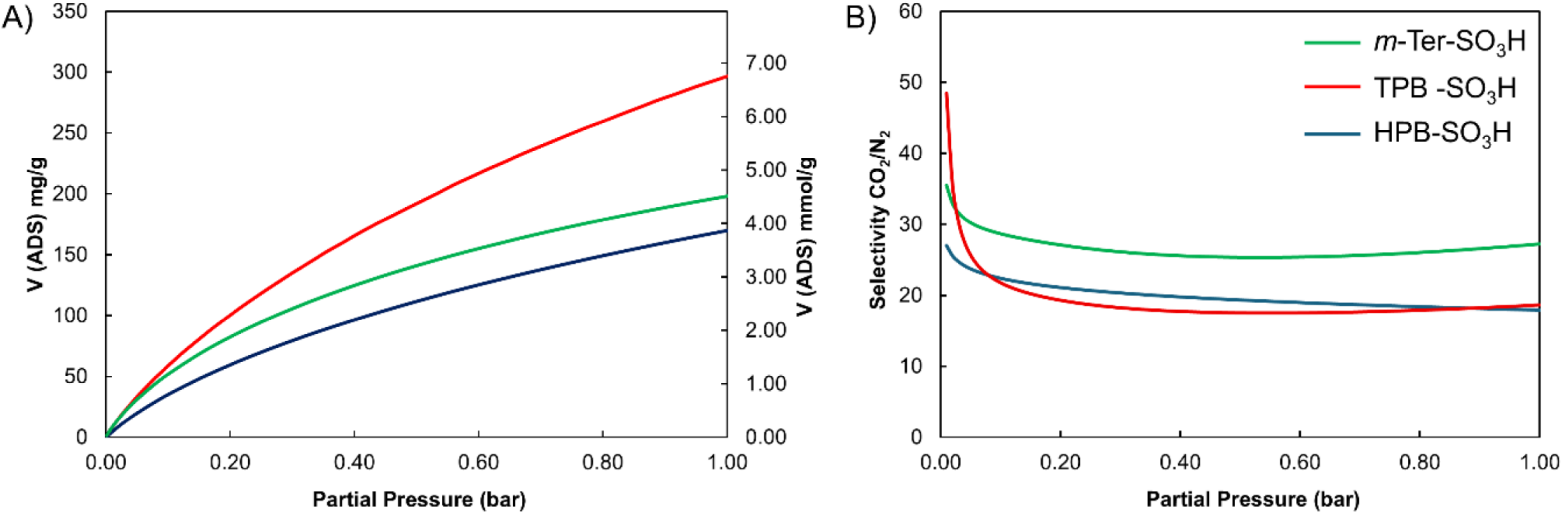
(A) Isothermal CO_2_ adsorptions at 273 K for all reported polymers; (B) IAST CO_2_/N_2_ selectivity for a hypothetical 15/85 CO_2_/N_2_ mixture.

Despite its slightly lower CO_2_ uptake, the polymer exhibited the highest CO_2_/N_2_ selectivity within the series, with a value of 27.8 under simulated postcombustion conditions (15/85 CO_2_/N_2_ ratio at 298 K). This highlights its promising potential as a cost-effective material for carbon capture applications.^32^ The pore size distribution (PSD) was determined from CO_2_ adsorption measurements at 273 K and calculated using NLDFT, yielding results consistent with typical HCP-PIMs, with three main pore populations in the range 3.5–9 Å.^23^ A closer examination of the ***m*** **-TER** polymers (Figure S15) reveals that the hydrocarbon form contains a higher fraction of pores in the 5–9 Å range, whereas the sulfonated derivative exhibits a more pronounced peak at 3.5 Å. This suggests that the introduction of sulfonic groups increases the proportion of ultramicropores, which in turn accounts for the enhanced CO_2_/N_2_ selectivity of these materials. Similar behavior is observed across all sulfonated polymers (Figure S16).

To investigate the morphology of the polymers and to enable a comparison between hydrocarbon and sulfonated HCPs, SEM imaging was carried out for all reported HCP materials. As shown in Figure 3, the TBP-based polymers, both hydrocarbon and sulfonated, showed a similar globular morphology with relatively large particle sizes, consistent with our previous findings.^23^ The *m*-Ter polymers also displayed aggregated domains, although the hydrocarbon derivative appeared more plate-like compared to its sulfonated counterpart. The most pronounced differences were observed in the HPB polymer, which presented a highly amorphous structure with significantly smaller particle sizes. This morphology is particularly advantageous for heterogeneous catalysis, as the increased surface area provides a greater number of accessible active sites for substrate interactions.^33,34^ Images with other magnifications can be found in Figures S32–S37.

**Figure 3:**
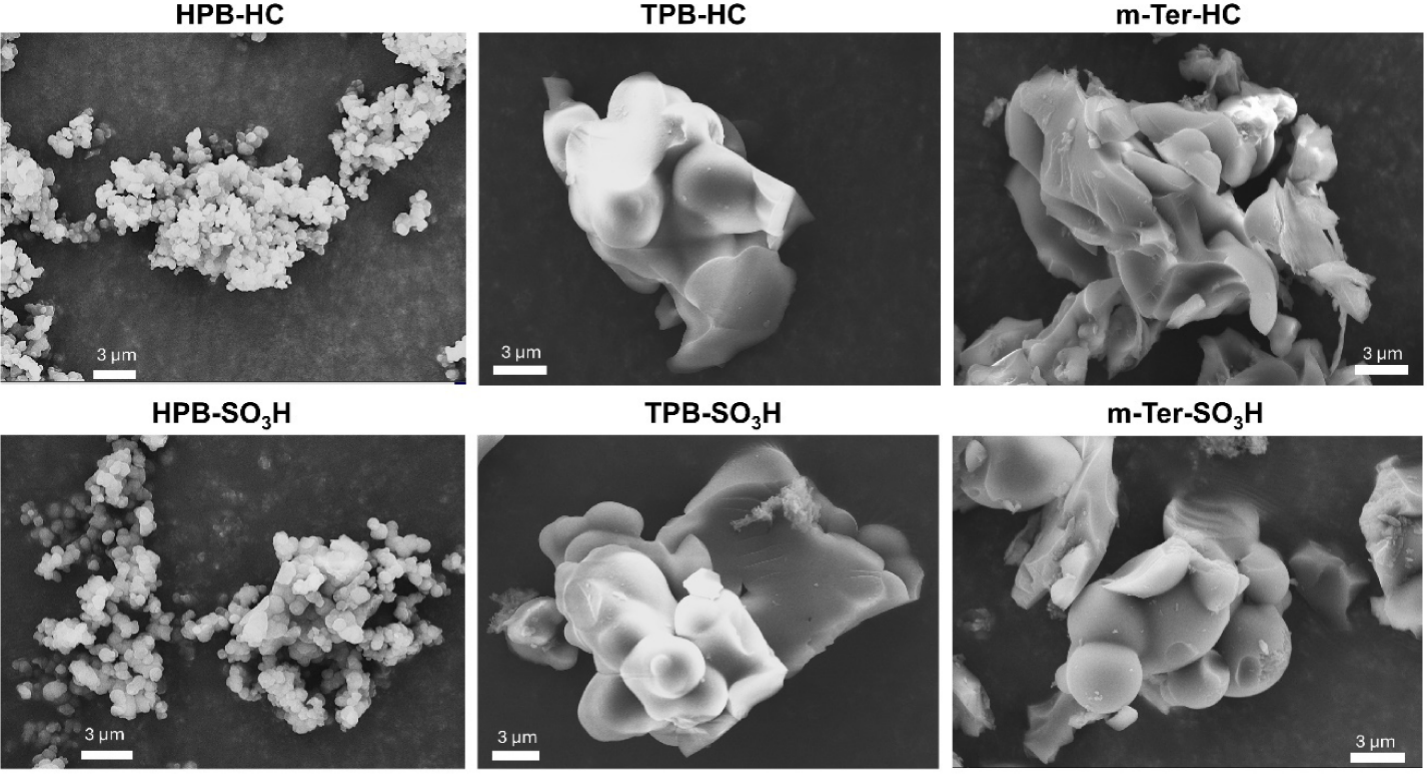
SEM images of all reported HCP-polymers.

### Catalysis Results

In view of the high porosity of the materials and the presence of sulfonic moieties, we explored their potential in the acid-catalyzed synthesis of biodiesel. Sulfonated polymers are particularly appealing in this context, as materials bearing sulfonic groups are widely employed to enhance biodiesel production, and thus allow for direct comparison with similar systems reported in the literature.^35^ We began by studying the simple acid-catalyzed esterification of fatty acids, followed by the more challenging, but also more industrially relevant, transesterification of triglyceride oils, especially testing our results using waste cooked oils, such as sunflower oil.^36–38^

### Esterification of Fatty Acids

It is common practice to evaluate the suitability of catalysts for biodiesel synthesis through simple Fischer esterification reactions between long-chain fatty acids and alcohols.^39^

A representative example is provided by Tantisriyanurak et al., who employed conjugated microporous polymers functionalized with sulfonic groups as catalysts for this reaction, which relies on strong acid functionalities to proceed efficiently (Figure 4).^21^ Given the structural similarities between their materials and ours, we adopted their testing procedure as a benchmark. In a typical experiment, 1 mmol of lauric acid was stirred with 2 mL of methanol, and 10 mg of catalyst was added to the reaction vessel. The mixture was then heated to 60 °C, with the conversion monitored hourly by sampling (∼40 μL) and analyzing the reaction progress via ^1^H NMR spectroscopy (Figure S18, with the explanation of how to interpret the spectra and so the conversion). The same tests were also conducted at room temperature, however, significantly lower conversions were observed, so a slightly elevated temperature was maintained for the experiments. To broaden the scope of the study and expand the range of substrates, we also evaluated the catalytic performance of our polymers with other common fatty acids, including palmitic, stearic, and oleic, which were chosen as they are commonly found in commercial oils. The main conversions are summarized in Table 2, while detailed kinetic data from hourly sampling are provided in Figure S17 and Table S1.

**Figure 4:**
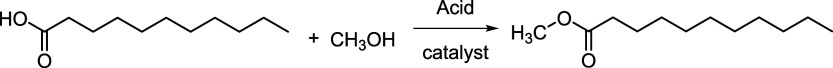
Fisher esterification of lauric acid.

**Table 2:** Conversion of Fatty Acids into the Corresponding Esters^a^

Catalysts	Acid used	Alcohol	Total acid density (mmol/g)	Conversion (% at 24 H)
***m*-Ter-SO**_**3**_ **H** (this work)	Palmitic	Methanol	2	95
	Lauric			95
	Oleic			96
	Stearic			95
**TPB-SO**_**3**_ **H** (this work)	Palmitic	Methanol	1.65	96
	Lauric			95
	Oleic			95
	Stearic			96
**HPB-SO**_**3**_ **H** (this work)	Palmitic	Methanol	1.76	98
	Lauric			Quantitative
	Oleic			Quantitative
	Stearic			Quantitative
Sulfonated murumuru-shell^40^	Oleic	Methanol	4.2	97
Sulfonated carbon spheres^41^	Oleic	Methanol	6.3	91
SO_3_H-KSC^35^	Palmitic	Methanol	14.3	92
s-BG + DEB^21^	Lauric	Methanol	9.11	93.6
s-CERs^42^	Palmitic	Methanol	5	99
SO^3^H-ZnAl_2_O_4_ ^43^	Oleic	Methanol	4.17	75

a Reaction conditions: 1 mmol of fatty acid, 2 mL of methanol, 10 mg of catalyst, 60 °C. All referenced catalytic tests were carried out at temperatures ≥ 60 °C.

A detailed analysis of our data demonstrates that our polymers are highly competitive in comparison to previously reported materials, particularly considering that their total acidity (i.e., the number of active sites) is somewhat lower than that of similar polymers.

Most experiments show that the reactions reach near-completion, with conversion to methyl esters ≥ 95%, and in the specific case of **HPB-SO**_**3**_ **H** 100% for 3 out 4 acids, within 24 h; however, reaction progress (Figure S17 and Table S1) reveals that the majority of conversion occurs rapidly within the first 6–8 h, after which the reaction rate slows down, and a plateau is observed. This rapid initial activity underscores the efficiency of our materials despite their moderate acidity, with the sulfonated **HPB** and **TPB** HCPs achieving the highest conversions, although the considerably less expensive ***m*** **-TER** polymer also demonstrates impressive catalytic performance. Considering the effect of the porosity for this reactions, it is evident that although the catalytic performance with different fatty acids is broadly comparable across the series (see especially Figure S17 and Table S1), the more microporous polymers such as **TBP-SO**_**3**_ **H** and **HPB-SO**_**3**_ **H** achieve faster and higher conversions than the slightly less porous ***m***-TER, which nonetheless remains a highly competitive catalyst, also because it demonstrates a slightly higher total acidity (Table 2).

### Transesterification of Triglyceride Oils

After establishing the efficient conversion of fatty acids into their corresponding esters, we employed the same polymer catalysts for the more demanding, but commercially more relevant, transesterification of triglycerides. In this case, commonly available oils such as sunflower, coconut, sesame, and rapeseed oils were used as substrates (structures shown in Figure S25). Transesterification involves the reaction of the triglycerides present in these oils with methanol, producing long-chain fatty acid methyl esters (FAMEs), the main components of biodiesel, along with glycerol as a byproduct (Figure 5). The relative simplicity of the transesterification reaction, combined with the abundance and sustainability of its feedstocks, makes it a highly attractive pathway for future fuel production. In this work, we initially employed readily available commercial oils, purchased directly from supermarkets and used without further purification, as practical substrates for small-scale laboratory testing.

**Figure 5:**
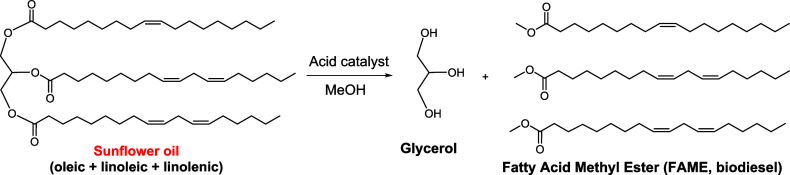
Transesterification of sunflower oil by HCP-SO_3_H catalysts.

Using such materials provided a reliable indication of catalytic performance under realistic conditions. Nevertheless, there is ongoing debate about the ethics of producing biodiesel from edible oils, since crops devoted to biofuel production could instead contribute to alleviating global hunger. This concern is particularly relevant to so-called first-generation biofuels so, more sustainable approaches now focus on waste biomass or algae, which require significantly less land and avoid competition with food supplies.^8^ In line with these considerations, and to strengthen the relevance of our study, we also tested the same catalytic reactions using waste cooking oils, demonstrating that the process remains effective with nonedible, recycled feedstocks. As with the fatty acid conversions, the progress of the transesterification reactions was monitored by ^1^H NMR spectroscopy. Although the spectra of oils are considerably more complex than those of simple fatty acids, evaluating the conversions proved less difficult than anticipated. Despite the higher number of resonances associated with the oils, the key signals are relatively well resolved and allow for reliable quantification. A representative example of this analysis, together with a detailed explanation, is provided in Figure S19. To better demonstrate the correct production of FAMEs, ^1^H NMR spectra of the catalytic reaction products obtained after 24 h for the different oils and, as a typical example, with TBP-SO_3_H as a catalyst, have been included in the (Supporting Information Figures S20–S24). Table 3 summarizes the performance of our hyper-cross-linked sulfonated polymers in this important transesterification reaction. All materials showed good catalytic activity across the range of tested oils, with **TBP-SO**_**3**_ **H** and **HPB-SO**_**3**_ **H** achieving faster conversions than ***m*** **-TER-SO**_**3**_ **H**, which nevertheless remained a competitive catalyst.

**Table 3:** Conversion of Triglyceride Oils into the Corresponding (FAME) Esters^a^

Catalysts	Oil used	Total acid density (mmol/g)	Conversionat 24 H (%)	Time to reach ∼90% Conversion (H)
*m*-Ter	Sesame	2	95	18
	Coconut		89	21
	Sunflower		95	17
	Rapeseed		92	21
TPB	Sesame	1.65	98	12
	Coconut		98	15
	Sunflower		98	17
	Rapeseed		98	15
HPB	Sesame	1.76	98	12
	Coconut		98	15
	Sunflower		98	12
	Rapeseed		98	15
PVA-99-SSA^36^	Soybean	1.19	92	>24
GR-SO_3_H^37^	Palm	na	98	14
Bagasse-derived catalyst^44^	Palm	4.11	94.8	6
ACPhSO_3_H^45^	Rapeseed	0.98	95	7
s-BG + DEB^21^	Coconut	9.11	98	24
MOF-5 ^38^	Waste cooking	na	82	12

a Reaction conditions: 100 mg of oil, methanol (5.7 mL) and catalyst (60 mg), 60 °C. All referenced catalytic tests were carried out at temperatures ≥ 60 °C.

In all cases, apart from ***m*** **-TER-SO**_**3**_ **H** with coconut oil, conversions equal or larger than 95% were reached after 24 h. Sunflower and sesame oils were converted more rapidly (∼90% after 12 h with **HPB-SO**_**3**_ **H**), whereas reactions involving coconut oil, and particularly rapeseed oil, proceeded more slowly, reflecting the influence of substrate structure and size on the reaction kinetics. This can be attributed to two factors: rapeseed oil contains the largest triglyceride molecules among those tested, while coconut oil is fully saturated and exhibits a very low solubility in methanol,^46^ both of which likely contribute to slower reaction rates.^47^

The results shown in Tables 2 and 3 demonstrate that the synthesized polymers exhibit catalytic performance comparable to, and in several cases surpassing, previously reported systems. Glišić and Skala have reported that the production of FAME from triglycerides is accelerated by the enhanced solubility of the substrates in the reaction medium. For this reason, some research groups deliberately add fatty esters to the reaction mixture to improve conversion rates.^48^ Another approach to increase the reaction efficiency involves employing more extreme conditions, such as supercritical methanol, which has been shown to significantly speed up transesterification, often in combination with cosolvents to further enhance substrate accessibility.^46^ As observed with the fatty acid experiments, the microporous polymers **TBP-SO**_**3**_ **H** and **HPB-SO**_**3**_ **H** also delivered superior performance with the oils compared to ***m*** **-TER-SO**_**3**_ **H**, confirming that microporosity plays a key role in facilitating these transesterification reactions (Figure 6). More detailed performance data are provided in Table S2.

**Figure 6:**
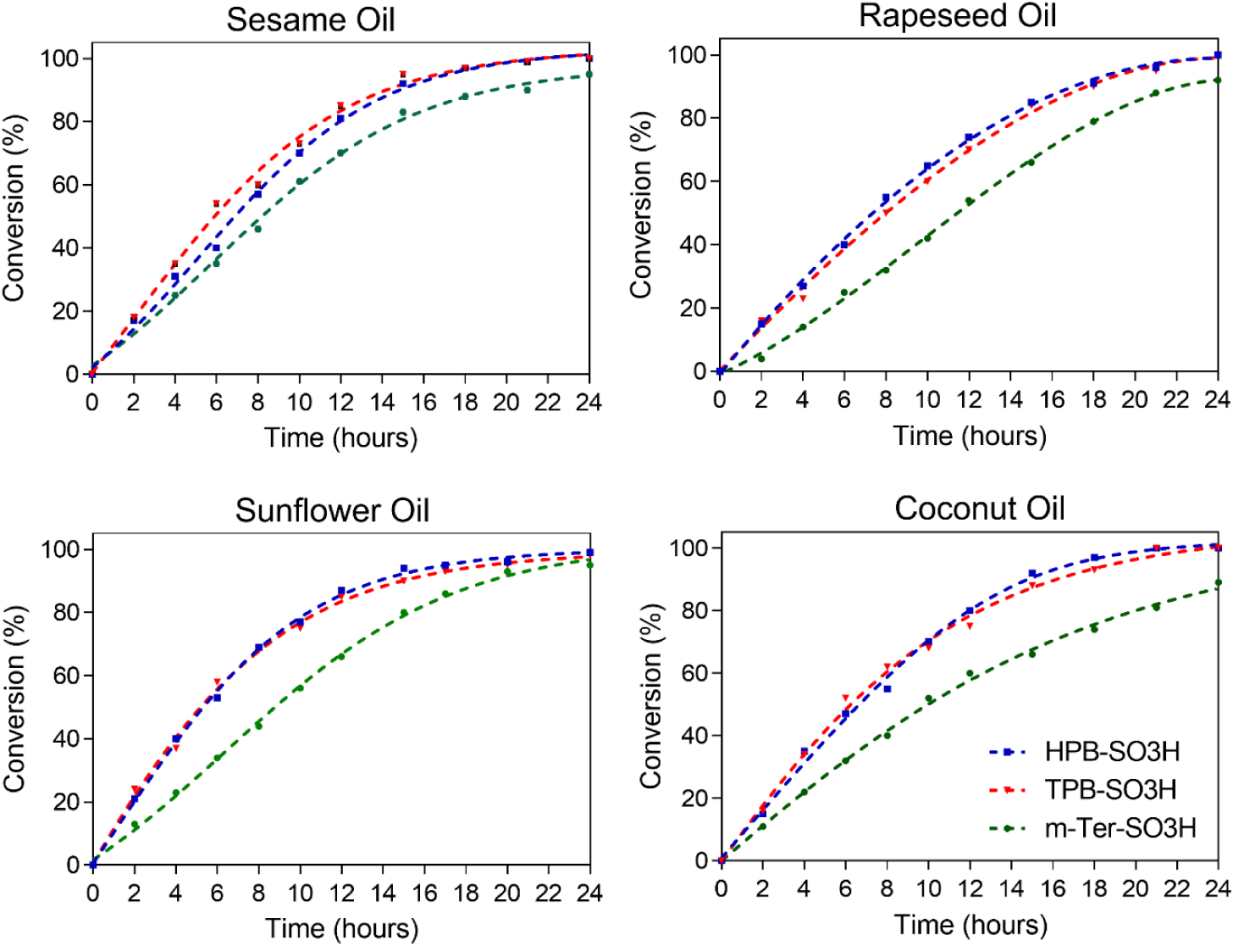
Hourly progress of the conversion of triglyceride oils into the corresponding (FAME) esters.

Although nitrogen and CO_2_ adsorption suggests that the pore size of microporous polymers is very small, experimental evidence of catalytic activity with both acids and bulky/oily substrates indicates that reagents can still access internal sites. This can be rationalized by a “*sneaking-in*” effect, in which polymer chain mobility and solvent-induced swelling transiently enlarge pore windows, permitting slow diffusion of molecules that are nominally too large. Similar *gate-opening* or *breathing phenomena* have been extensively reported in even less flexible porous materials such as MOFs and COFs, where linker motions or adsorption-induced transitions enable guest ingress beyond the apparent pore cutoff, despite slowing down the diffusion and so the initial kinetic.^49,50^

In the case of sulfonated microporous polymers, this dynamic porosity represents a clear advantage over nonporous analogues that also contain sulfonic acid moieties, since enhanced internal accessibility increases the density of catalytically active sites available to reagents.^51^ To further evaluate the efficiency of the catalysts, we investigated the effect of reducing both the catalyst loading and the amount of methanol used in the transesterification reaction. Figure 7 shows the reaction progress of sunflower oil with **TPB-SO**_**3**_ **H** under these varied conditions. Remarkably, when the volume of methanol was reduced from the standard 5.7 to 1.9 mL, a high conversion was still achieved. This result is particularly significant because methanol not only acts as a reagent but it is also known to facilitate the dissolution of the FAME products while the reaction progresses.^46^ Therefore, maintaining high conversion under reduced solvent conditions not only highlights the intrinsic efficiency of the catalyst but also minimizes solvent waste, thereby enhancing the overall sustainability of the system.

**Figure 7:**
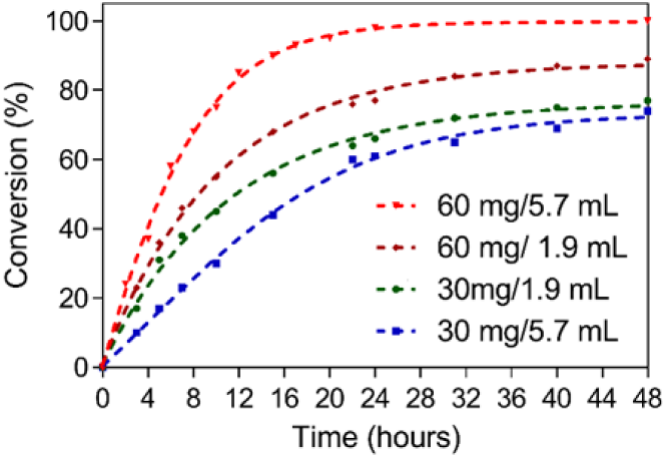
Hourly progress of catalysis run with different amounts of TPB-SO_3_H and methanol.

As expected, reducing the catalyst loading resulted in lower conversions. However, relatively good results were obtained when either catalyst or methanol amounts were reduced, compared to the case where reduced catalyst with excess methanol were used, highlighting the materials’ efficiency under solvent-limited conditions. The lowest performance was, in fact, observed when both the catalyst amount was halved and the methanol volume remained high, emphasizing the balance required between these two parameters. Overall, our materials proved to be highly efficient even under reduced solvent and catalyst conditions, highlighting their potential for more sustainable transesterification processes.

### Scale Up and Recycle Using Waste Sunflower Oil

To demonstrate that the polymer synthesis reported here is not only efficient but also scalable, a crucial factor for evaluating a practical catalyst, we increased the reaction scale from 1 to 10 g of the starting hydrocarbon monomer. The yield remained consistent and practically quantitative for both the Friedel–Crafts reaction that forms the hydrocarbon monomer and the subsequent sulfonation. Notably, less solvent was required at the larger scale, underlining both the efficiency and practicality of the process. These results confirm that the materials are not only cost-effective but also readily scalable for larger applications. To further evaluate catalyst performance and reusability, we investigated its recyclability in the transesterification of sunflower oil with **TPB-SO**_**3**_ **H** over multiple cycles (Figure 8).

**Figure 8:**
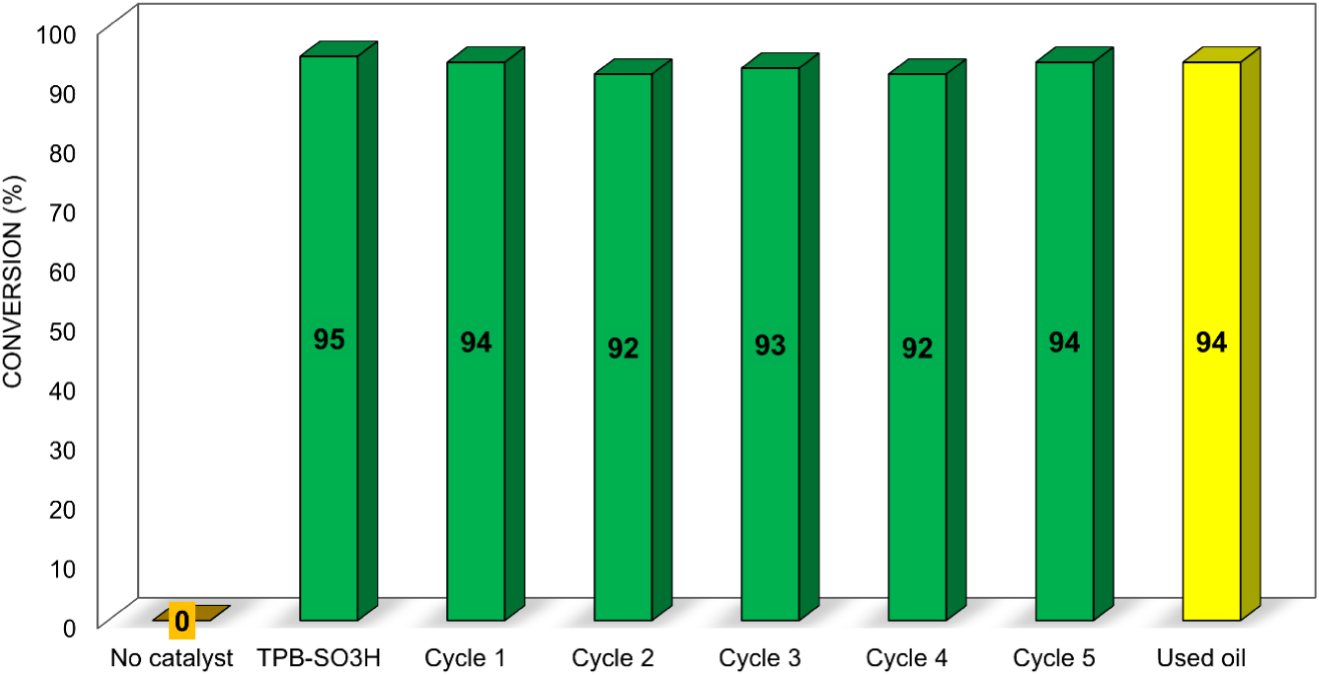
Recycling test of reaction with 60 mg of TPB-SO_3_H for 24 H and with used oil at 60 °C. In the case of no-catalyst, the 0% indicates that we did not detect any FAME production from a simple thermal reaction.

After each run, the catalyst was simply recovered by filtration, washed with methanol, dried, and reused. Conversions after 24 h remained essentially constant over five cycles, demonstrating stable activity. Remarkably, similarly high conversions were obtained using waste cooking sunflower oil, indicating that these catalysts are highly effective even with nonedible and impure feedstocks, highlighting their potential for sustainable and practical applications.

## Conclusion

In this work, we have demonstrated that hyper-cross-linked sulfonated polymers, structurally related to PIMs, function as highly efficient and versatile catalysts for the production of FAME biodiesel. By systematically varying the monomer composition to increase the content of aromatic moieties, we obtained a series of microporous polymers with tailored textural properties. These materials were thoroughly characterized, not only to assess their surface area and porosity, but also to evaluate their potential in applications such as carbon capture and CO_2_/N_2_ separation. The catalytic performance of these polymers was rigorously tested in two complementary reactions: Fischer esterification of free fatty acids and transesterification of triglyceride oils derived from the same types of fatty acids. This dual evaluation highlights the broad applicability of the catalysts, showing that their efficiency is not limited to simple fatty acid substrates but extends to complex, naturally occurring triglycerides. In both reactions, the polymers achieved nearly quantitative FAME conversions under standard conditions and retained impressive activity even when both catalyst and methanol loadings were reduced, emphasizing their intrinsic efficiency and robustness. Scalability and recyclability were further demonstrated, confirming that the catalysts can be applied in larger-scale processes without loss of performance. Critically, the polymers also maintained high activity when using waste cooking sunflower oil, demonstrating that efficient biodiesel production is achievable from nonedible, low-cost feedstocks. Overall, this study establishes a clear structure–property–performance relationship, linking polymer architecture and porosity to catalytic efficiency across both esterification and transesterification reactions. The combination of high activity, feedstock versatility, reusability, and scalability positions these hyper-cross-linked sulfonated polymers as a compelling platform for sustainable biodiesel production, while offering a broader framework for the design of next-generation porous catalysts for diverse chemical transformations. We are confident that process intensification, such as operation under continuous-flow conditions, will further enhance mass transfer and reaction efficiency, leading to significantly shorter reaction times.

## Supplementary Material



## Notes

Materials and methods; synthesis and characterization of polymers; TGAs; adsorption studies; catalytic studies; tables; various figures; solid-state ^13^C NMRs (PDF)
