# Supplementary material for: Hyper-Cross-Linked Microporous Polymers as Cheap and Efficient Catalysts for the Synthesis of Biodiesel
Source: ACS Appl Eng Mater. 2026 Mar 25;4(4):1841–51. doi: 10.1021/acsaenm.6c00096 (PMC13122583; doi:10.1021/acsaenm.6c00096)
Supplement: Supplementary file 1 [file em6c00096_si_001.pdf]

Supporting information

Hypercrosslinked Microporous Polymers as Cheap and Efficient Catalysts for the Synthesis of Biodiesel

C. Grazia Bezzu, [a] * *Natasha Hawkins,* [a] *Rebecca Foster,* [a] *Ariana R. Antonangelo,* [a] *James W Ryan,* [a] *Anna Williamson* [a] *and Mariolino Carta* [a.b] *.

[a] Department of Chemistry, Swansea University, Faculty of Science and Engineering, Singleton Park, Swansea, SA28PP, UK.

[b] Instituto de Síntesis Química y Catálisis Homogénea, CSIC-Universidad de Zaragoza, C/Pedro Cerbuna 12, Facultad de Ciencias, Zaragoza 50009, Spain

Email: c.g.bezzu@swansea.ac.uk; mariolino.carta@swansea.ac.uk. mariolino.carta@csic.es.

Contents

General methods and equipment 2
Experimental part 3
HPB-HC[3] 3
HPB-HSO3[1] 3
TPB-HC[2] 4
TBP-SO3H[2] 4
m-TER 4
m-TER-SO3H 5
Catalysis tests 5
Catalysis tests: Esterification of Acids 5
Catalysis tests: Trans-esterification of oils 5
TGA of polymers 6
Adsorption data 7
Pore size distributions 13
^{1}H NMR assessment of esterification of Lauric acid with TPB-SO3H 15
^{1}H NMR assessment of Trans-esterification of sunflower oil. 16

Conversion of oils to FAME 17
Composition of oils 22
^{13}C MAS SSNMR 23
SEM images 26
FT-IR of Polymers 32
References 33

General methods and equipment

Commercially available reagents and gases were used without further purification. The oils employed (refined edible oils, including commercially available sunflower oil, coconut oil, sesame oil and rapeseed oil) are characterized by low free fatty acid contents, typically below 0.5 wt%, which is consistent with the maximum acid value of 0.5 mg KOH g^{-1} specified for biodiesel in international standards (EN 14214, ASTM D6751). **Coconut oil** and **Sesame oil** were purchased from Thermo Scientific (product 10169841 and product 10157453, respectively). **Sunflower oil** and **rapeseed oil** were purchased from a local grocery shop. All reactions using air/moisture sensitive reagents were performed in oven-dried or flame-dried apparatus, under a nitrogen atmosphere. TLC analysis refers to analytical thin layer chromatography, using aluminium-backed plates coated with Merck Kieselgel 60 GF254. Product spots were viewed either by the quenching of UV fluorescence, or by staining with a solution of Cerium Sulfate in aqueous H_2SO_4. Melting points were recorded using a Cole-Parmer Stuart™ Digital Melting Point Apparatus and are uncorrected. Low-temperature N_2 (77 K) and CO_2 (273 K and 298 K) adsorption/desorption measurements of polymer powders were made using an Anton Paar Nova600. Samples were degassed for 480 min at 80 °C under high vacuum prior to analysis. The data were analyzed with the software provided with the instrument. NLDFT analyses were performed to calculate the pore size distribution and volume, considering a carbon equilibrium transition kernel at 273 K based on a slit-pore model; the kernel is based on a common, one center, Lennard-Jones model. TGAs were performed using the device PerkinElmer STA 6000 at a heating rate of 10 °C/min from 30 to 1000 °C. ATR Infrared spectra were measured in the solid state using a PerkinElmer Spectrum Two FT-IR Spectrometer and acquired in the range 4000-500 cm^{-1}. ^{1}H NMR spectra were recorded in the solvent stated using an Avance Bruker DPX 500 (500 MHz) instruments, with ^{13}C NMR spectra recorded at 125 MHz. Solid-state ^{13}C NMR spectra were recorded using a Bruker Avance III spectrometer equipped with a wide-bore 9.4 T magnet (Larmor frequencies of 100.9 MHz for ^{13}C). Samples were packed into standard zirconia rotors with 4 mm outer diameter and rotated at a magic angle spinning (MAS) rate of 12.5 kHz. Spectra were recorded with cross polarization (CP) from 1H using a contact pulse (ramped for 1H) of 1.5 ms. High-power ($\nu_1 \approx$ 100 kHz) TPPM-15 decoupling of 1H was applied during acquisition to improve resolution. Signal averaging was carried out for 6144 transients with a recycle interval of 2 s. Chemical shifts are reported in ppm relative to $(CH_3)_4Si$ (TMS) using the CH_3 signal of L-alanine (δ = 20.5 ppm) as a secondary solid reference. Scanning electron microscope (SEM) images were recorded using a ZEISS Crossbeam 550 field-emission scanning electron microscope (FE-SEM). Images were acquired using an accelerating voltage of 10 kV and a secondary electron detector (SED). The polymer powders were mounted on conducting carbon tape and coated with a thin layer of gold palladium (approximately 6-8 nm) using a sputter coater to minimize charging during imaging.

Experimental part

The synthesis and functionalization of hypercrosslinked polymers, apart from the novel ***m*-TER** were reproduced according to our previously reported procedure but scaled up.[1-2]

HPB-HC[3]

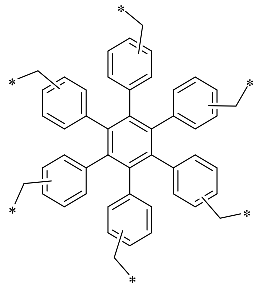

HPB (10.0 g, 18.7 mmol) and $AlCl_3$ (24.9 g, 187 mmol) were added to DCM (500 mL) and stirred at reflux for 24 hours under a nitrogen atmosphere. The solution was filtered, and the obtained powder washed with plenty of water and ethanol. The powder was washed sequentially via reflux with ethanol, chloroform, THF, acetone, and methanol, and finally dried in a vacuum oven at 100 ^{0}C for 20 hours. The HPB-network polymer (13.0 g, 21.2 mmol, ~100 %) was analysed by IR spectroscopy, TGA, and BET. SA_{BET} = 1932 m^2g^{-1}, TGA: Thermal degradation commences at 295 ˚C. FTIR-ATR (cm^{-1}): 1096, 1452, 2978. ^{13}C MAS SSNMR (101 MHz) δ (ppm) 136.69, 131.03, 126.72, 82.72, 72.49, 65.76, 54.44, 40.27, 34.15, 13.10.

HPB-HSO$_3$[1]

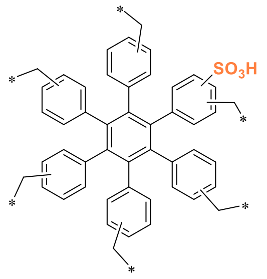

HPB-HC (5.0 g, 8.16 mmol) was added to H_2SO_4 (100 mL), stirred for 30 minutes, and heated to 60 °C for 8 hours under a nitrogen atmosphere. The solution was cooled to room temperature, poured over ice, and filtered. The solid was washed via heating to 60 °C in deionised water twice and filtered until the filtrate showed a neutral pH. The solid was refluxed in methanol twice, filtered, and dried in a vacuum oven at 60 °C for 18 hours. The obtained dark powder was analysed (5.58 g, 8.16 mmol, ~100 %; the yield is considered for a single sulfonic group, but the titration shows 1.16 per repeat unit). SA_{BET} = 1404 m^2g^{-1}, TGA: Thermal degradation commences at 165 ˚C. FTIR-ATR (cm^{-1}): 1039, 1175, 2980, 3420. ^{13}C MAS SSNMR (101 MHz) δ (ppm) 255.64, 235.97, 192.27, 135.92, 131.84, 72.44, 55.35, 34.28, 14.61.

TPB-HC[2]

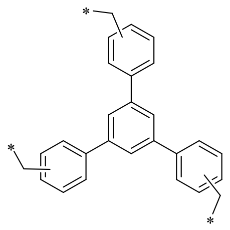

TPB (10.0 g, 32.6 mmol) and $AlCl_3$ (43.5 g, 326 mmol) were added to DCM (500 mL) and stirred at reflux for 24 hours under a nitrogen atmosphere. The solution was filtered, and the obtained powder washed with plenty of water and ethanol. The powder was washed sequentially via reflux with ethanol, chloroform, THF, acetone, and methanol, and finally dried in a vacuum oven at 100 ^{0}C for 20 hours. (11.3 g, 32.6 mmol, ~100 %). BET = 2540 m^2 g^{-1}; FT-IR ν max (cm^{-1}) 2968, 1700, 1600, 1392, 1048, 872. ^{13}C MAS SSNMR (101 MHz) δ (ppm) 162.5, 139.4, 131.6, 56.9, 35.3, 14.7, -12.9.

TBP-SO$_3$H[2]

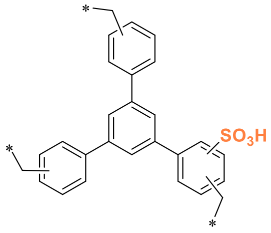

TPB-HC (5.0 g, 14.5 mmol) was added to H_2SO_4 (100 mL), stirred for 30 minutes, and heated to 60 °C for 8 hours under a nitrogen atmosphere. The solution was cooled to room temperature, poured over ice, and filtered. The solid was washed via heating to 60 °C in deionised water twice and filtered until the filtrate showed a neutral pH. The solid was refluxed in methanol twice, filtered, and dried in a vacuum oven at 60 °C for 18 hours. The obtained dark powder was analysed (5.3 g, 12.45 mmol, ~85 %). BET = 1585 m^2 g^{-1}, total pore volume =0.8521 (at P/P_0 = 0.9774); CO_2 adsorption at 273 K/1 bar = 298 mg g^{-1} (6.8 mmol g^{-1}); TGA: initial mass loss at 180 °C. FT-IR ν max (cm^{-1}) 2982, 1700, 1589, 1240, 1238, 1165, 1033, 606. ^{13}C MAS SSNMR (101 MHz) δ (ppm) 189.7, 138.0, 132.4, 79.6, 72.4, 69.5, 54.5, 48.5, 35.8, 15.2.

***m*-TER**

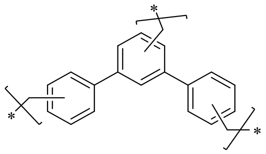

m-TER (10.0 g, 43.4 mmol) and $AlCl_3$ (57.9 g, 434 mmol) were added to DCM (500 mL). The mixture was refluxed for 24 hours under a nitrogen atmosphere. The solution was quenched into water, filtered, and washed with water. The solid was swelled in ammonia and filtered. Then

refluxed in ammonia for 20 minutes, filtered and washed with water. The obtained powder was then washed consecutively via reflux with ethanol, chloroform, THF, acetone and methanol, twice each with swelling of each solvent between each reflux. The brown-red powder was dried at 120 °C for 18 hours (13.2 g, 49.0 mmol, ~100 %). IR (cm^{-1}): 3014, 2884, 1700, 1600, 1435, 1403. TGA: Thermal degradation commences at: 303 °C. SA_{BET}: 1977m^2g^{-1}; Total pore volume = 1.190 (at P/P_0 ~ 0.98); ^{13}C MAS SSNMR (101 MHz) δ (ppm) 141.03, 132.05, 127.48, 58.40, 35.23, 16.30.

***m*-TER-SO_3H**

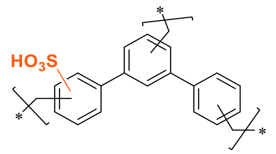

m-TER (5.0 g, 18.6 mmol) was added to H_2SO_4 (100 mL), stirred for 30 minutes, and heated to 60 °C for 8 hours under a nitrogen atmosphere. The solution was cooled to room temperature, poured over ice, and filtered. The solid was washed via heating to 60 °C in deionised water twice and filtered until the filtrate showed a neutral pH. The solid was refluxed in methanol twice, filtered, and dried in a vacuum oven at 60 °C for 18 hours. The obtained dark blue-black powder was analysed (5.9 g, 16.7 mmol, 90 %). IR (cm^{-1}): 3325, 2928, 1675, 1593, 1168, 1032, 892, 613. TGA: Thermal degradation commences at: 177 °C SA_{BET}: 1100 m^2g^{-1}; Total pore volume = 0.4975 (at P/P_0 ~ 0.98). ^{13}C MAS SSNMR (101 MHz) δ (ppm) 188.23, 139.09, 132.60, 53.92, 36.93, 18.25.

Catalysis tests

Reactions, for both esterifications and transesterifications, were carried out in glass tubes equipped with magnetic stirrer bars and fitted with water-cooled reflux condensers. The vessels were heated at 60 °C using a four-position temperature-controlled metal heating block filled with sand, allowing parallel reactions to be conducted under identical conditions with continuous stirring.

Catalysis tests: Esterification of Acids

1 mmol of acid, methanol (2 mL) and catalyst (10 mg) were stirred together at 60 °C. Samples (20 µL) were taken at regular intervals. Prior to measurement, excess methanol was removed from the sampled amount under a gentle stream of nitrogen, and the conversion was monitored via NMR ($CDCl_3$).

Catalysis tests: Trans-esterification of oils

100 mg of each oil, methanol (5.7 mL) and catalyst (60 mg) were stirred at 60 °C. Samples (20 µL) were taken at regular intervals. Prior to measurement, excess methanol was removed from the sampled amount under a gentle stream of nitrogen, and the conversion was monitored via NMR ($CDCl_3$).

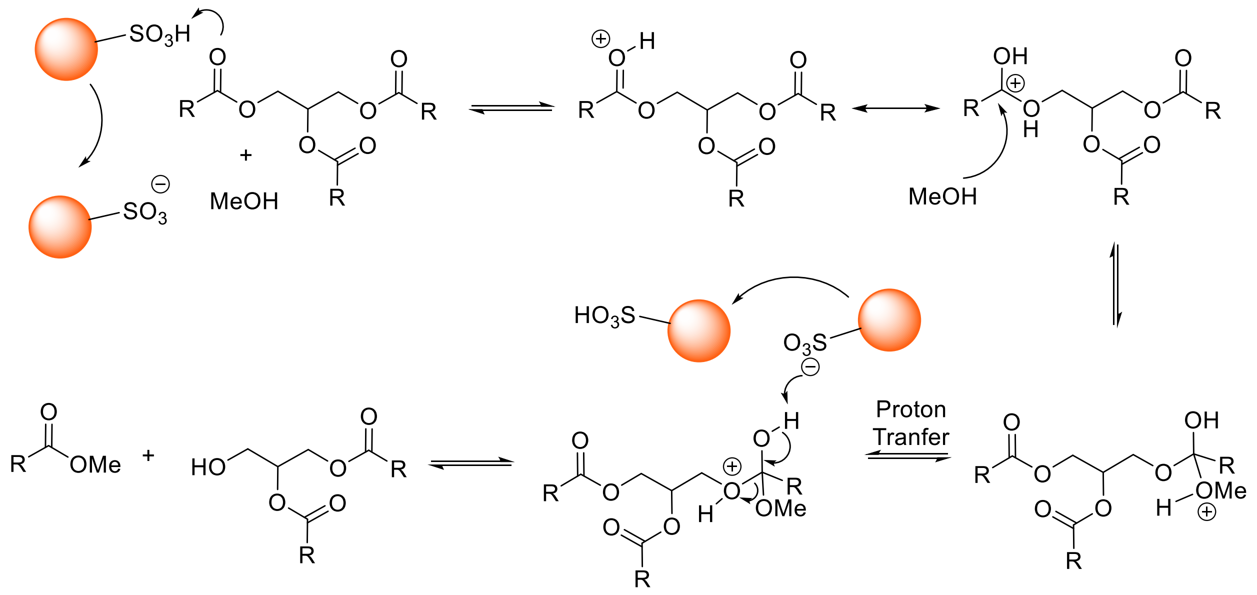

Scheme S1. Transesterification mechanism.

TGA of polymers

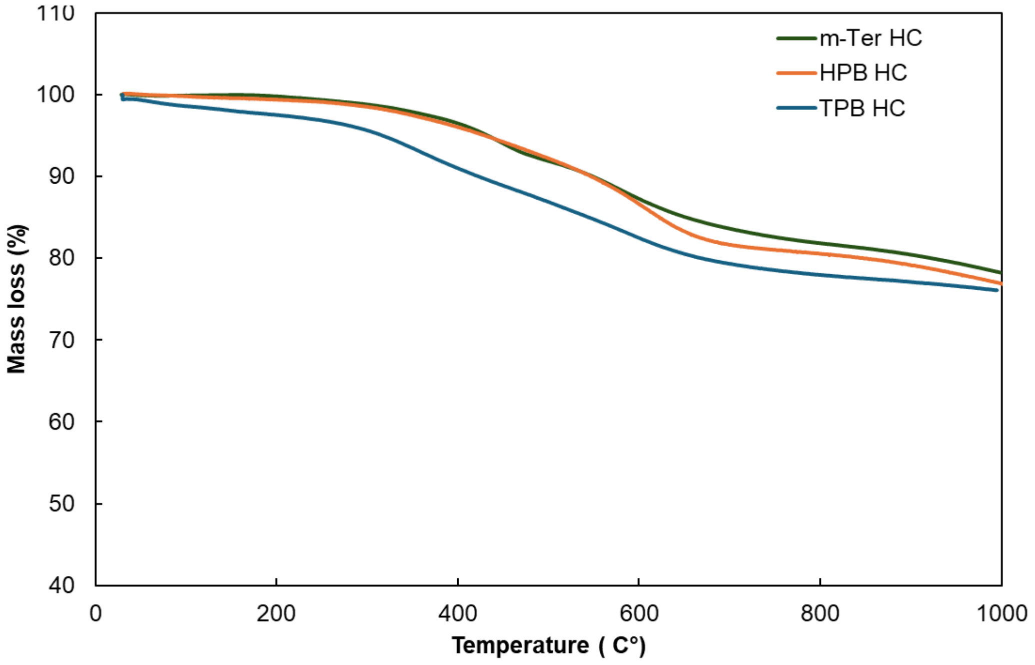

Figure S1. Overlay TGA of HC polymers

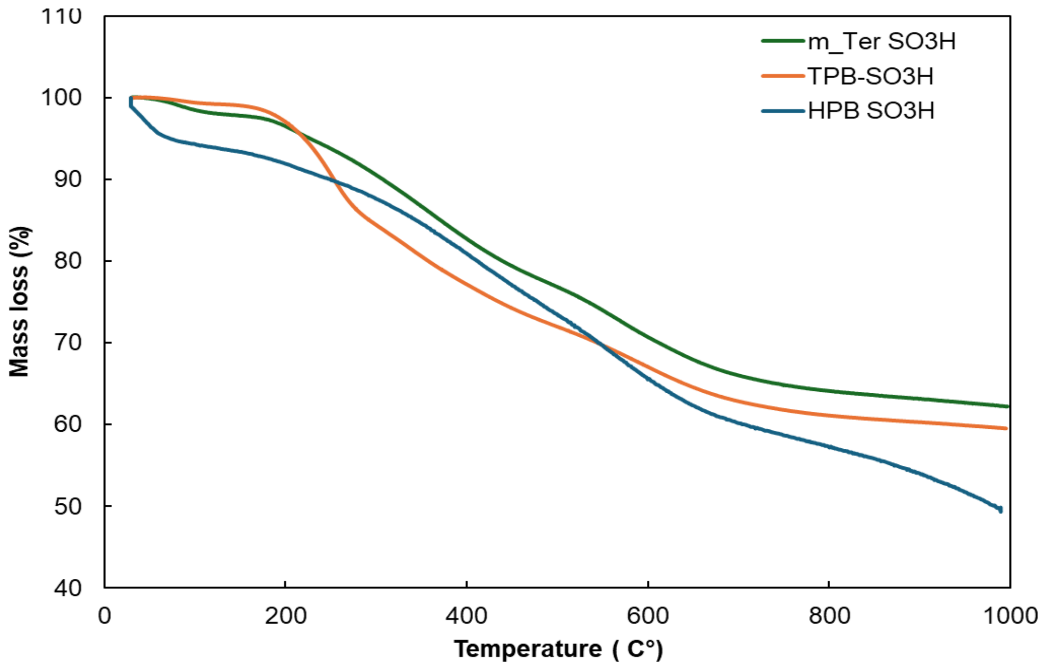

Figure S2. Overlay TGA of $-SO_3H$ polymers

Adsorption data

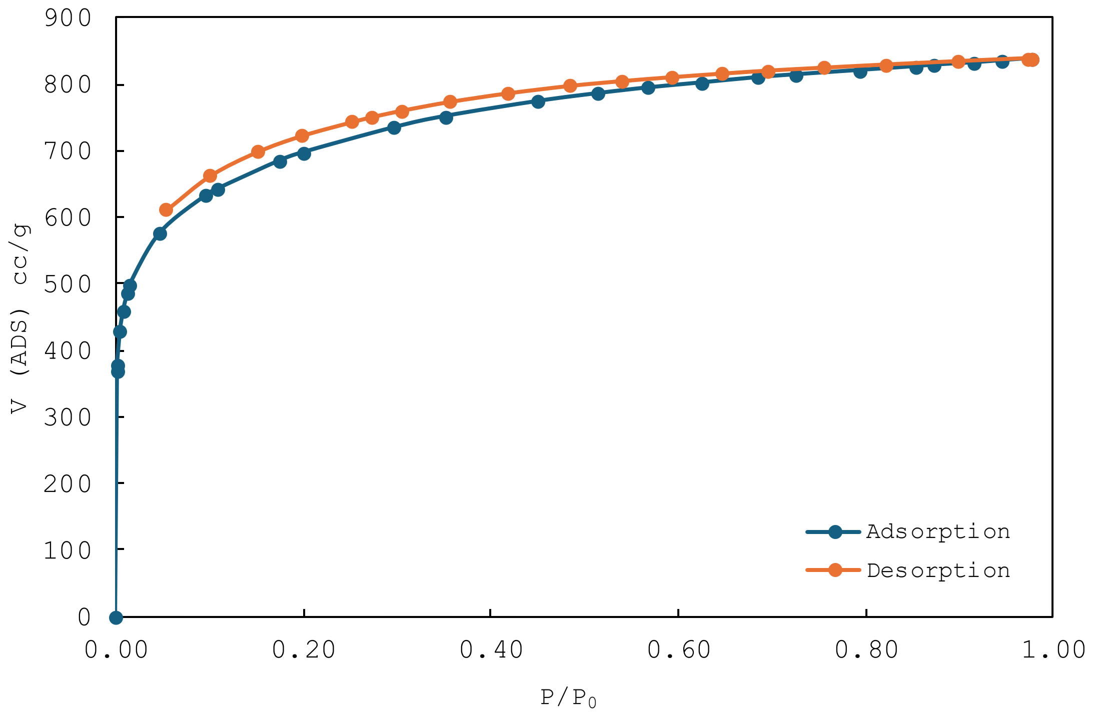

Figure S3. TPB HC N_2 @77 K

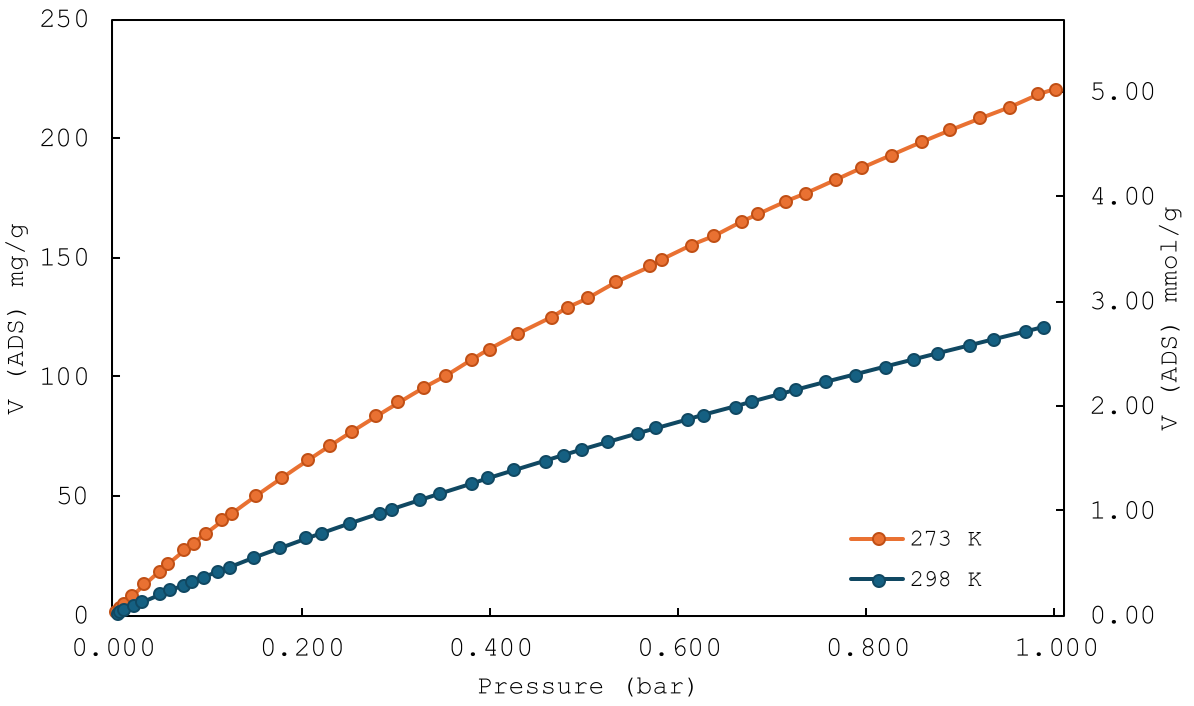

Figure S4. TPB HC CO_2 273 K and 298 K

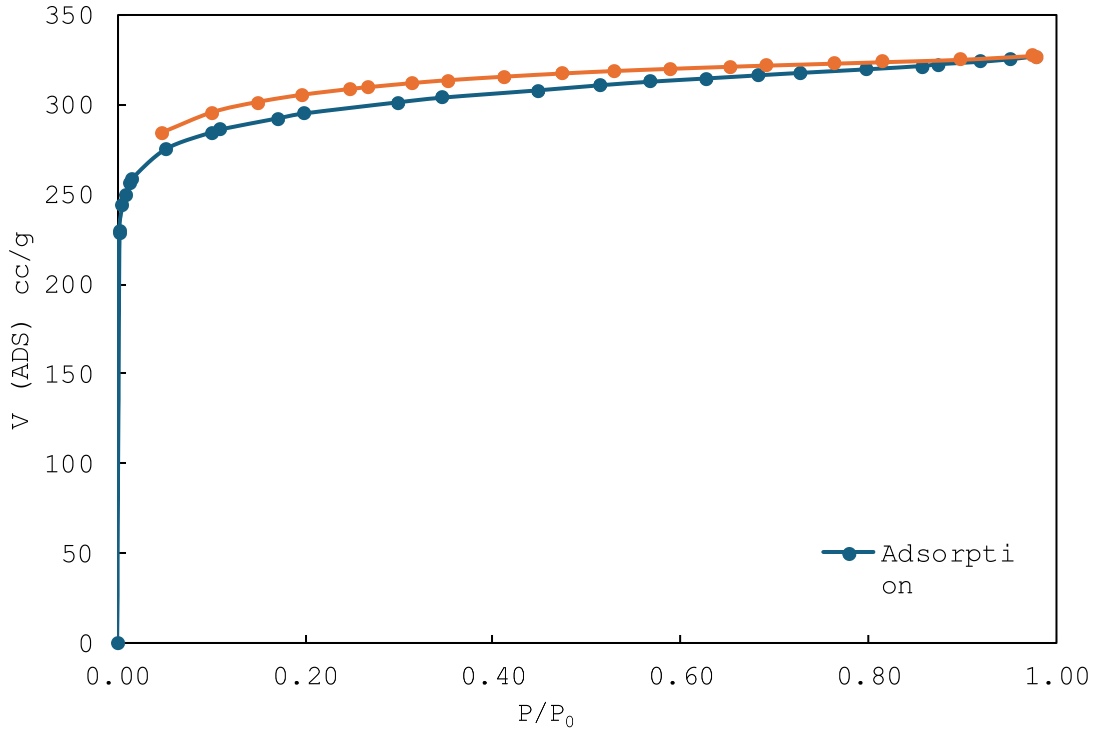

Figure S5. TPB -SO_3H N_2 77 K

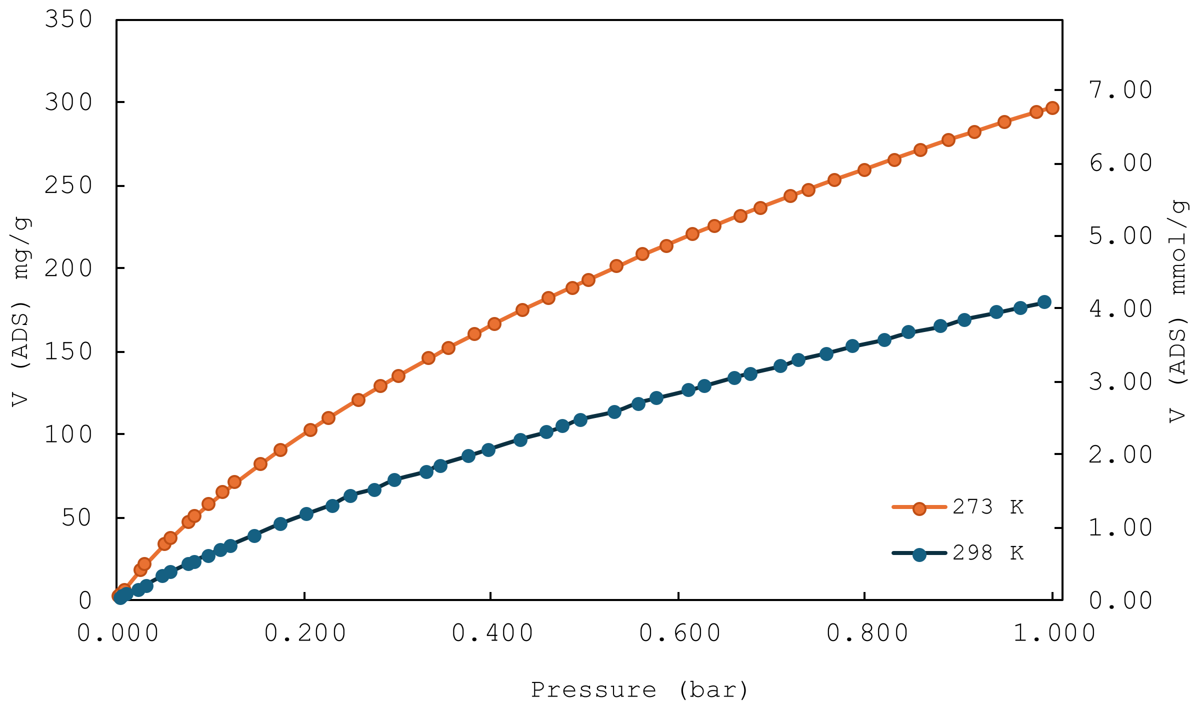

Figure S6. TPB -SO_3H CO_2 273 K and 298 K

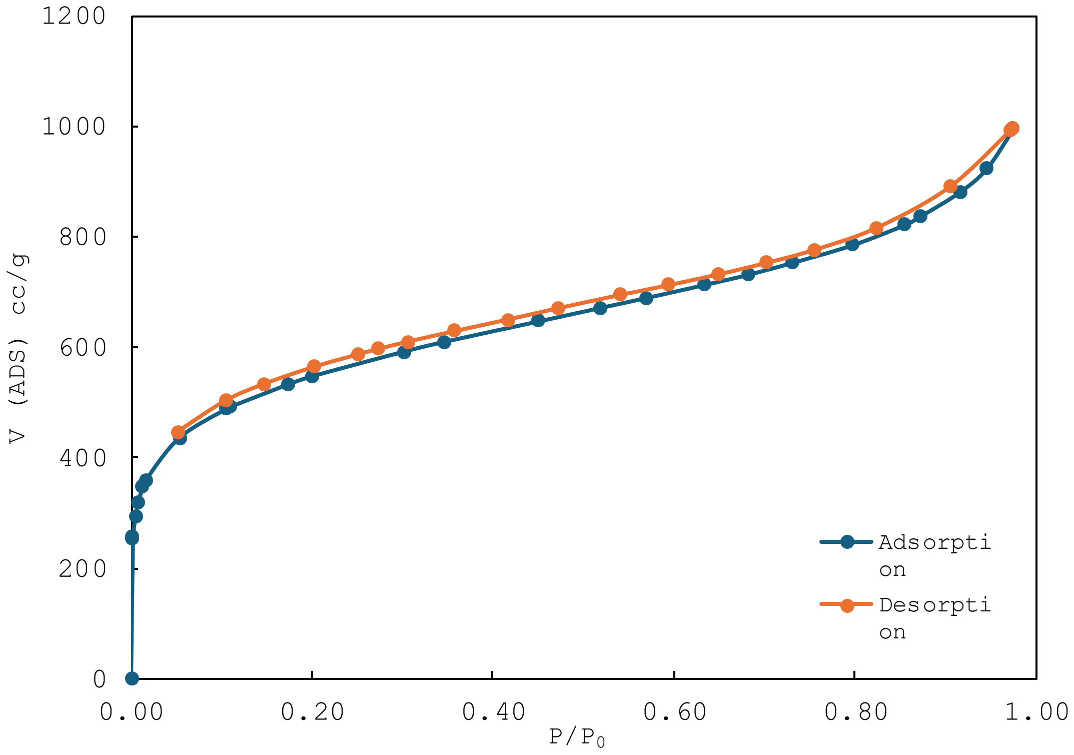

Figure S7. HPB HC N_2 @77K

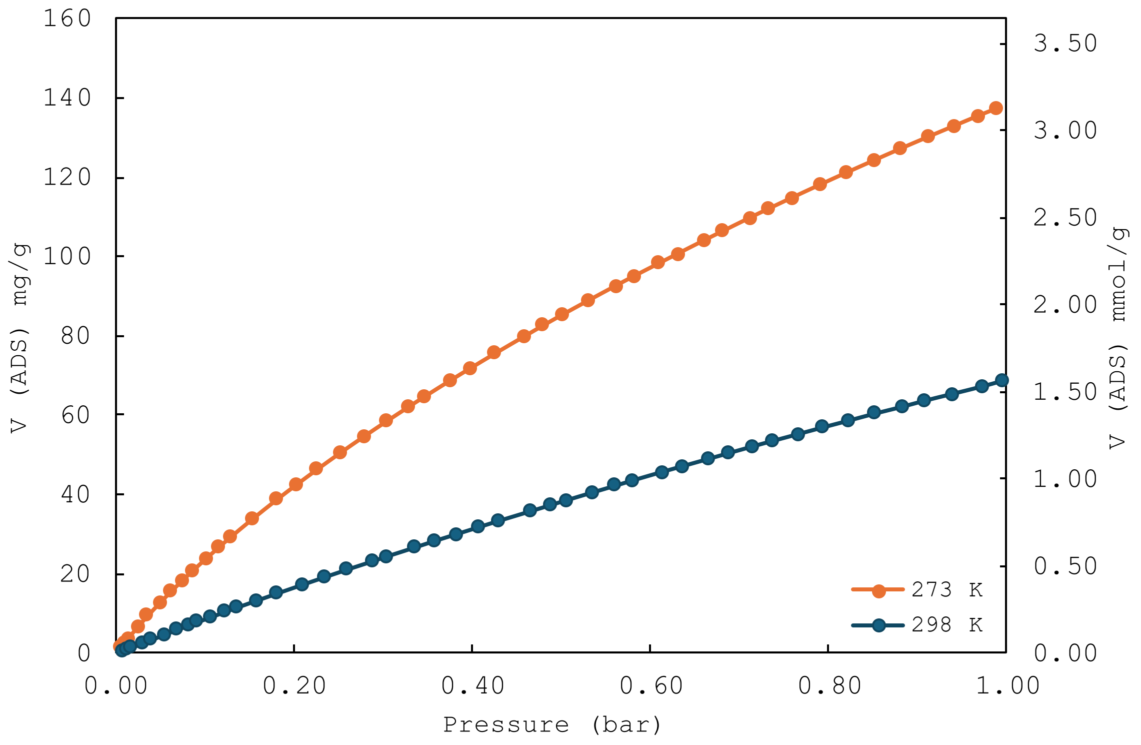

Figure S8. HPB HC CO_2 273 K and 298 K

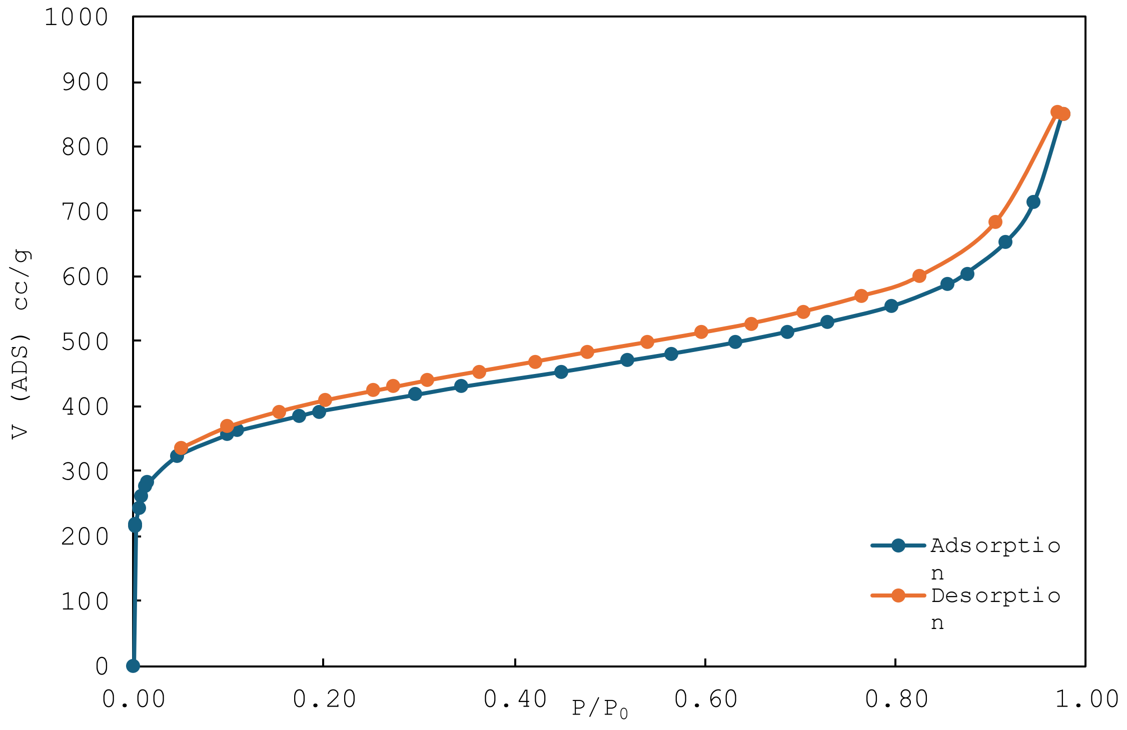

Figure S9. HPB-SO_3H N_2 @77 K

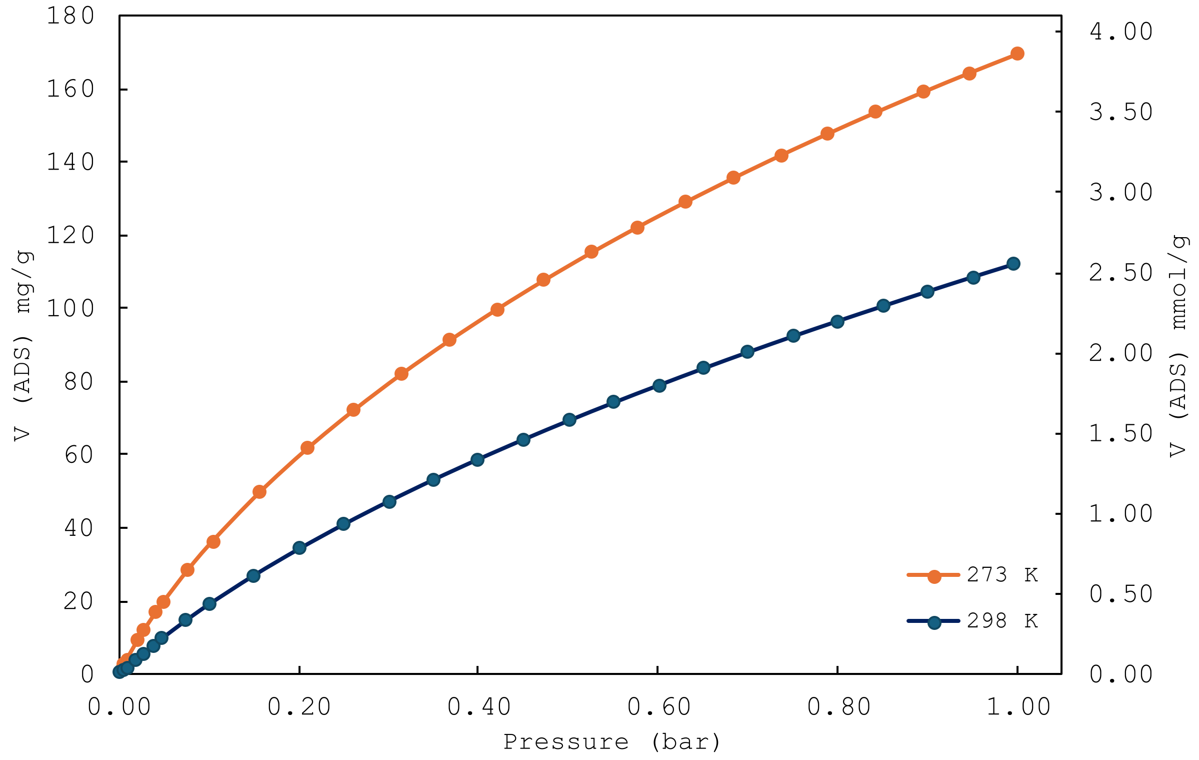

Figure S10. HPB-SO_3H CO_2 273 K and 298 K

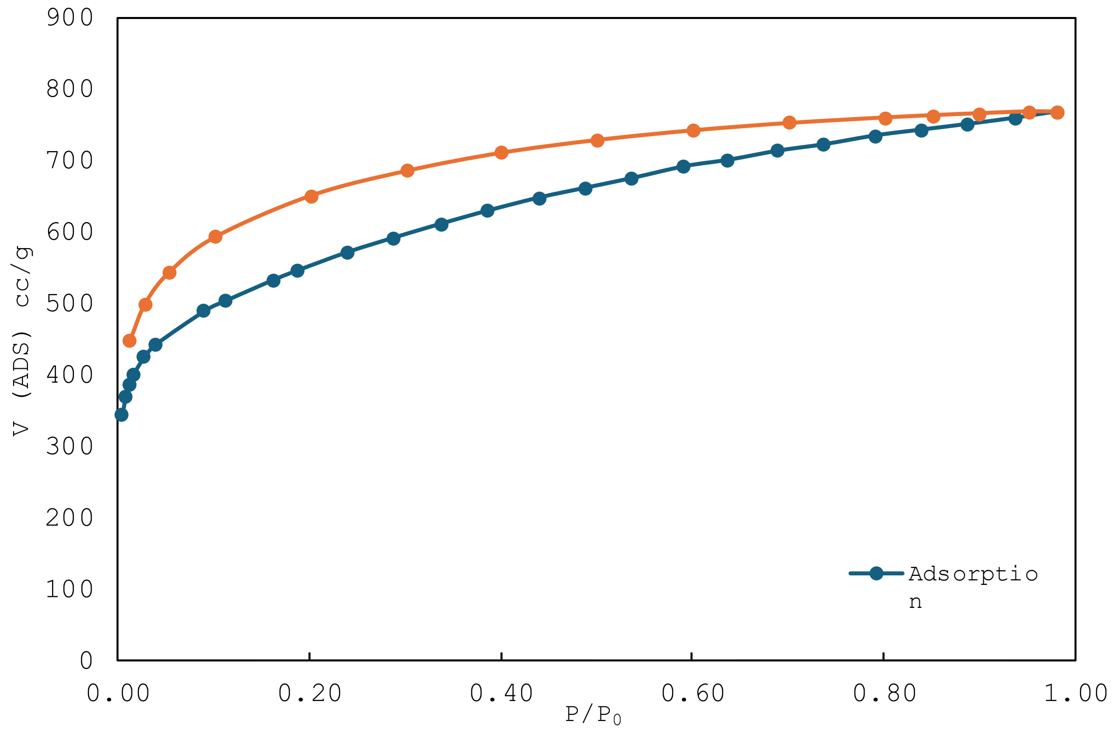

Figure S11. m-Ter-HC N_2 @ 77 K

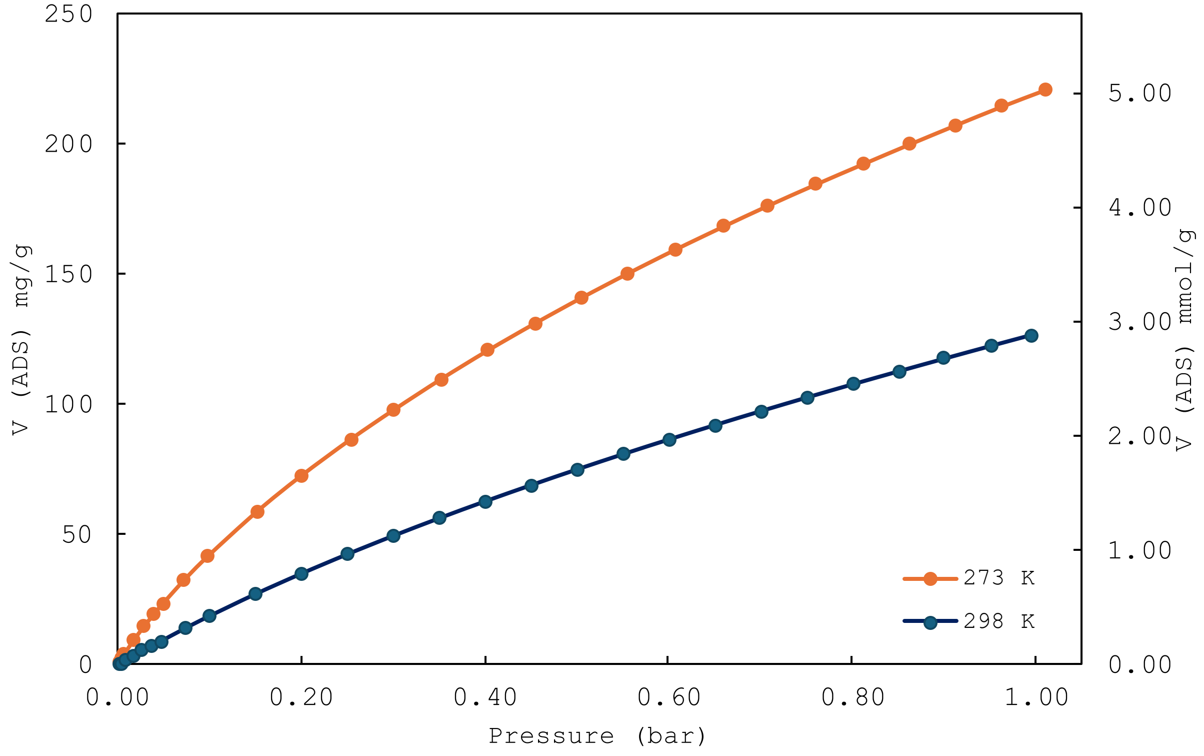

Figure S12. m-Ter HC CO_2 273 K and 298 K

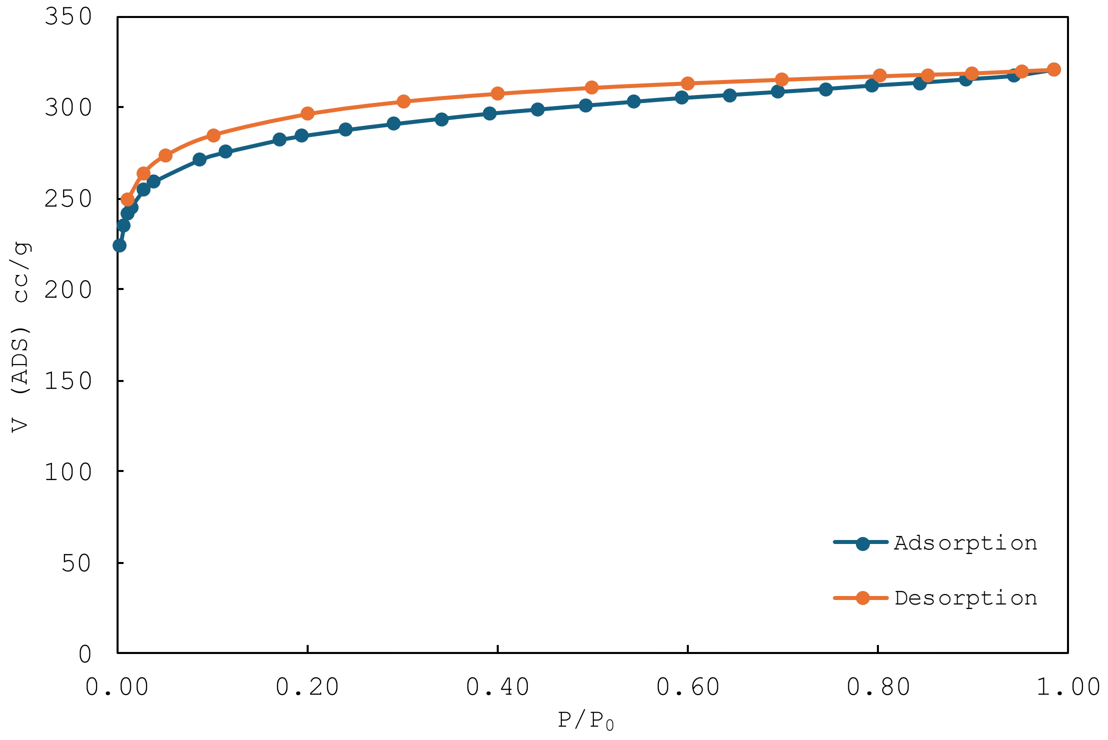

Figure S13. m-Ter-SO_3H N_2 @ 77 K

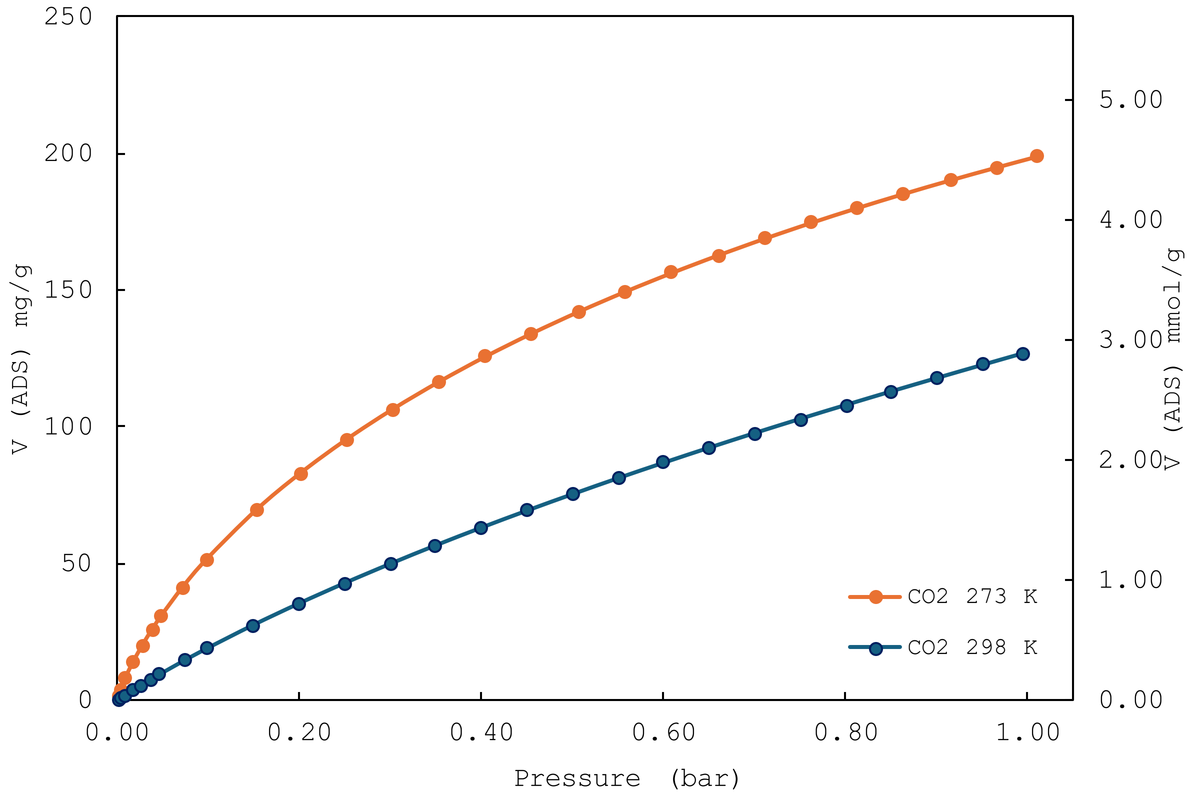

Figure S14. m-Ter-SO_3H CO_2 273 K and 298 K

Pore size distributions

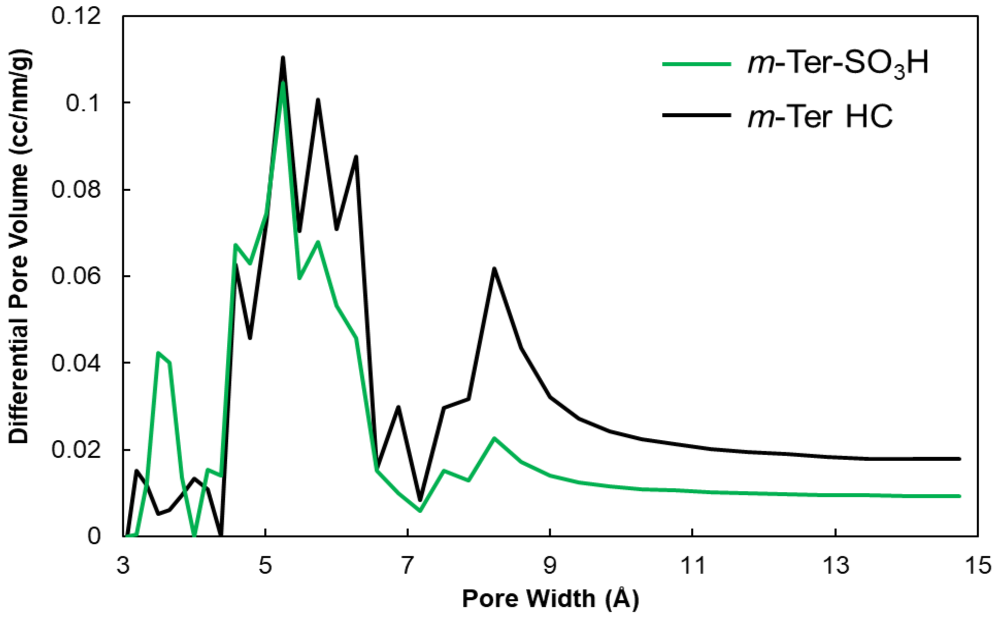

Figure S15. *m*-Ter-SO_3H vs *m*-Ter HC

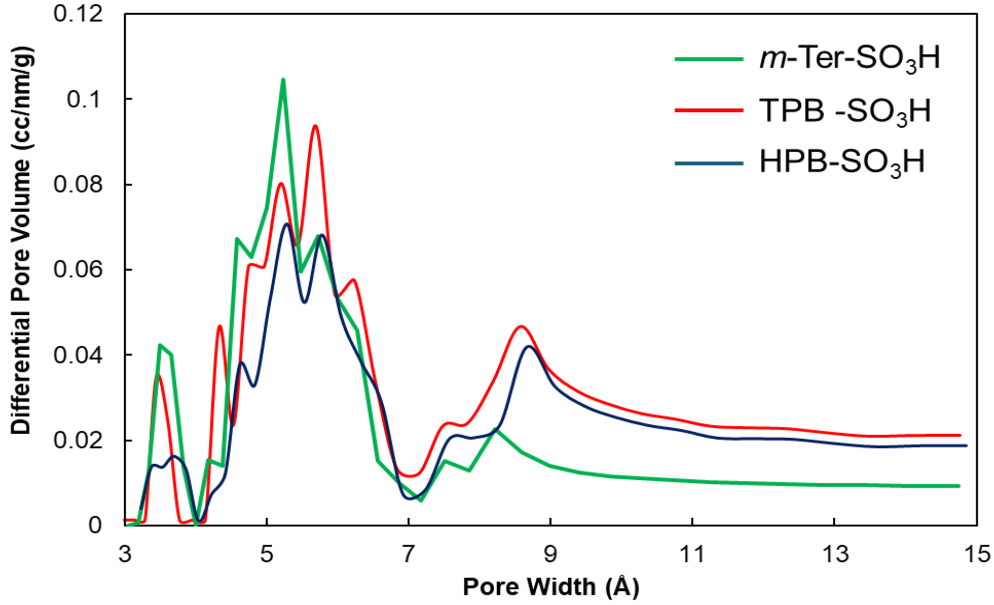

Figure S16. PSD of all HCP-SO_3H polymers reported in this work

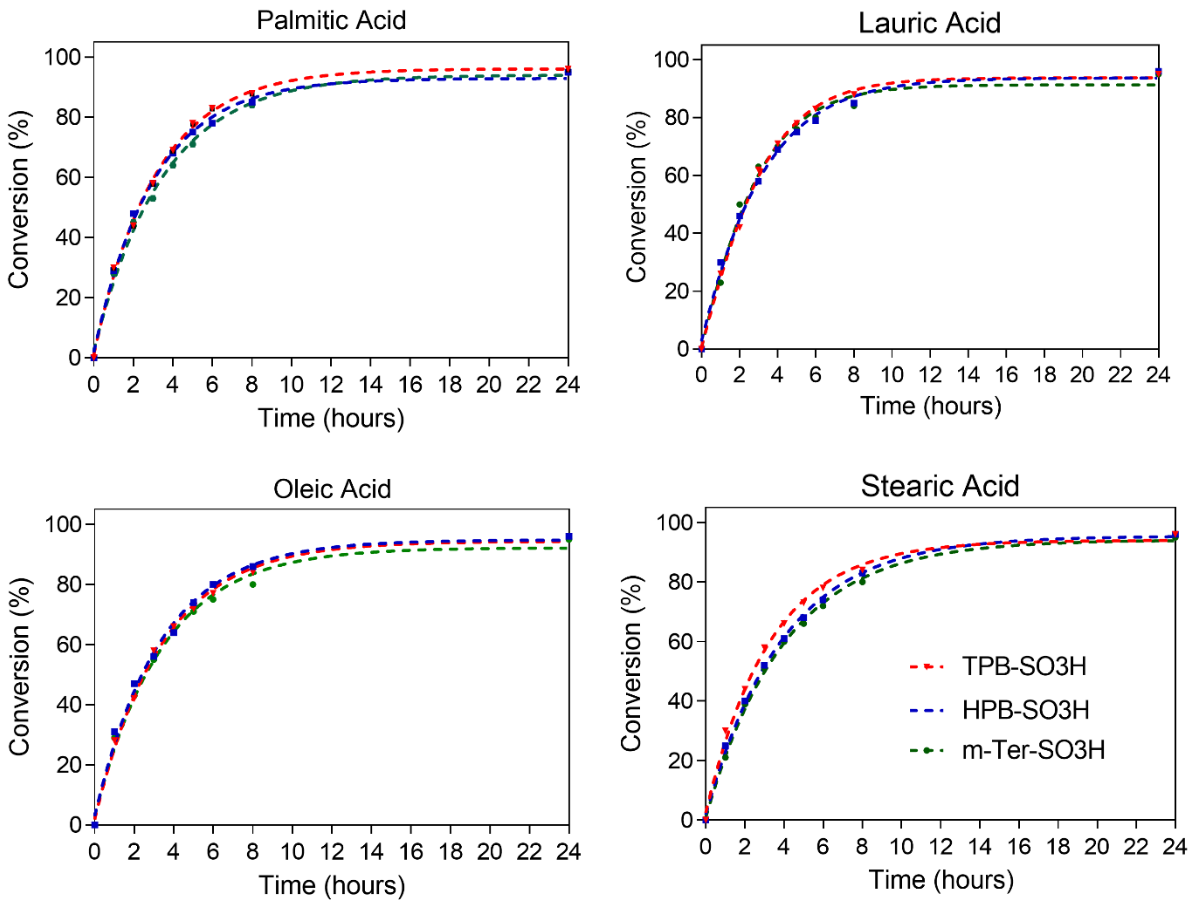

Figure S17. Conversion of fatty acids into esters.

^{1}H NMR assessment of esterification of Lauric acid with TPB-SO_3H.

Two key peaks are particularly informative during the reaction. To monitor conversion, we can track the signal at **2.35 ppm**, which in the lauric acid corresponds to the methylene group adjacent to the carbonyl. Upon ester formation, this peak shifts slightly upfield to **2.30 ppm**, reflecting increased shielding. When the reaction is incomplete, both signals are observed. In parallel, the methoxy peak of the ester at **3.66 ppm** gradually emerges, increasing from absent in the acid to fully present in the ester. By integrating these peaks, the relative amounts of reactant and product can be quantified, providing a direct measure of conversion and enabling comparison of catalyst performance.

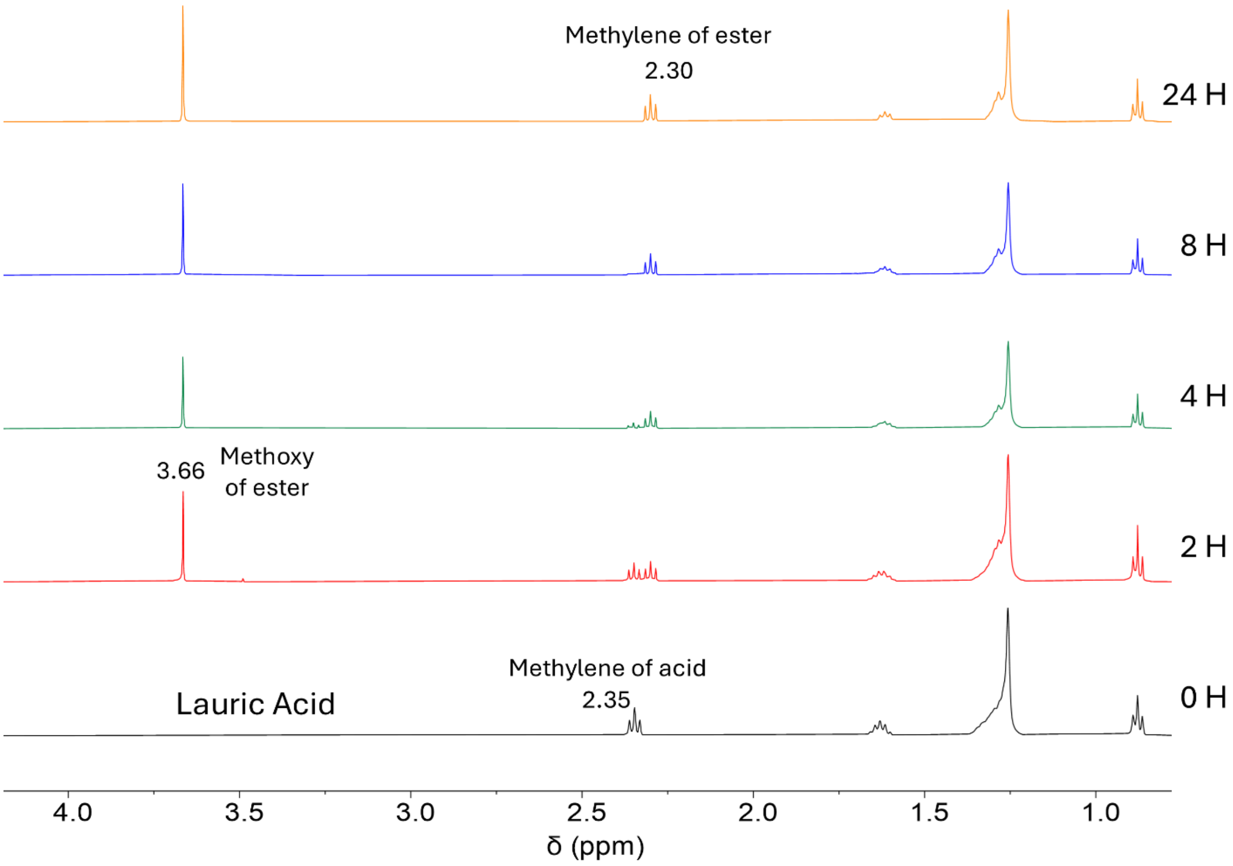

Figure S18: ^{1}H NMR of the evolution of the esterification of Lauric acid. Reagents and conditions: acid (1mmol), methanol (2 mL) and catalyst (TPB-SO_3H, 10 mg) at 60 °C.

^{1}H NMR assessment of Trans-esterification of sunflower oil.

The progression of the transesterification reaction can be monitored by **^{1}H NMR**, in a manner analogous to that described for the acid. The signals corresponding to the four methylene protons of the glyceridic group, centred at **4.29** and **4.14 ppm**, gradually diminish, while a new signal at **3.66 ppm**, characteristic of the methyl ester, appears. By integrating these peaks, the relative amounts of reactant and product can be quantified, providing a direct measure of conversion and enabling comparison of catalyst performance.

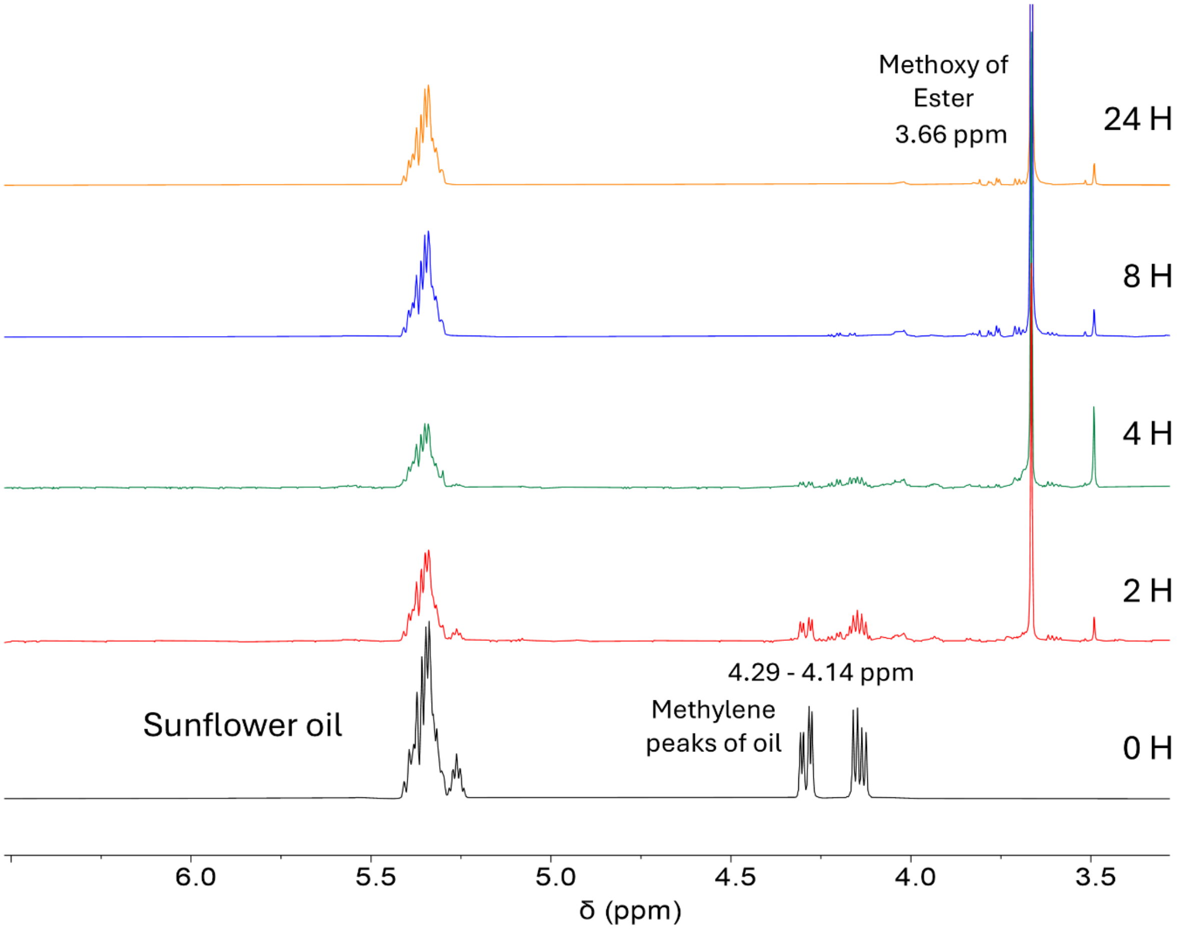

Figure S19: ^{1}H NMR of the evolution of trans-esterification of sunflower oil with TPB-SO_3H. Reagents and conditions: 100 mg of sunflower oil, methanol (5.7 mL) and catalyst (60 mg) were stirred at 60 °C. Samples (40 µL) were taken at regular intervals and the conversion was monitored via NMR ($CDCl_3$).

Conversion of oils to FAME

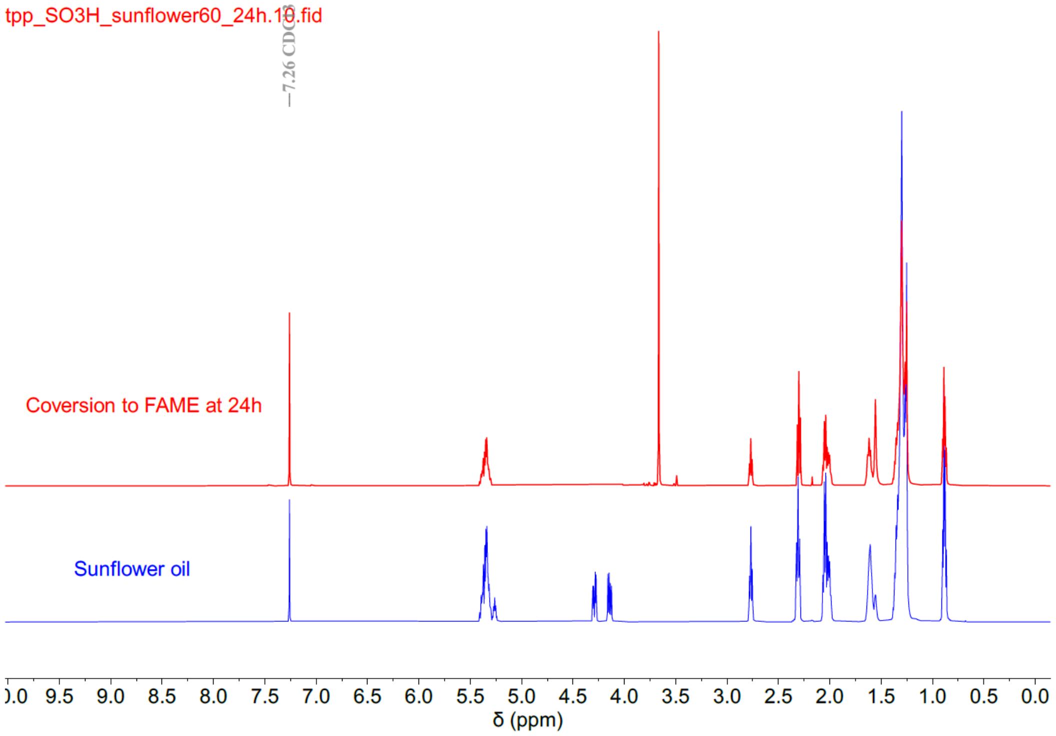

Figure S20: Conversion of sunflower oil to the correspondent FAME with TPB-SO_3H

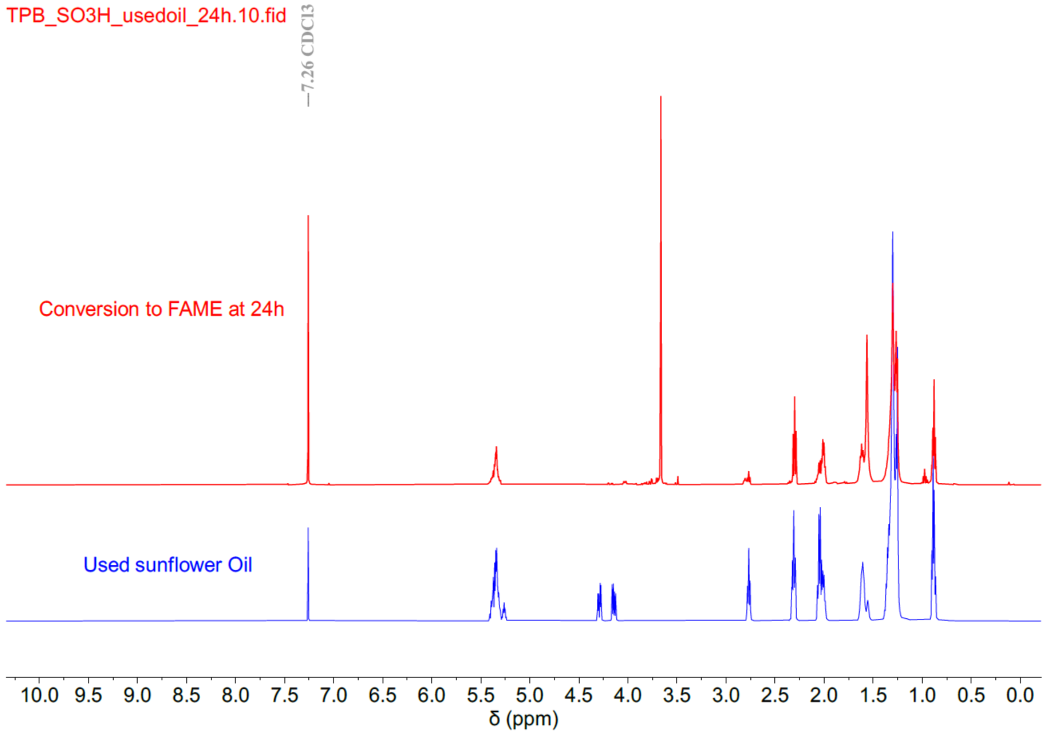

Figure S21: Conversion of used sunflower oil to the correspondent FAME with TPB-SO_3H

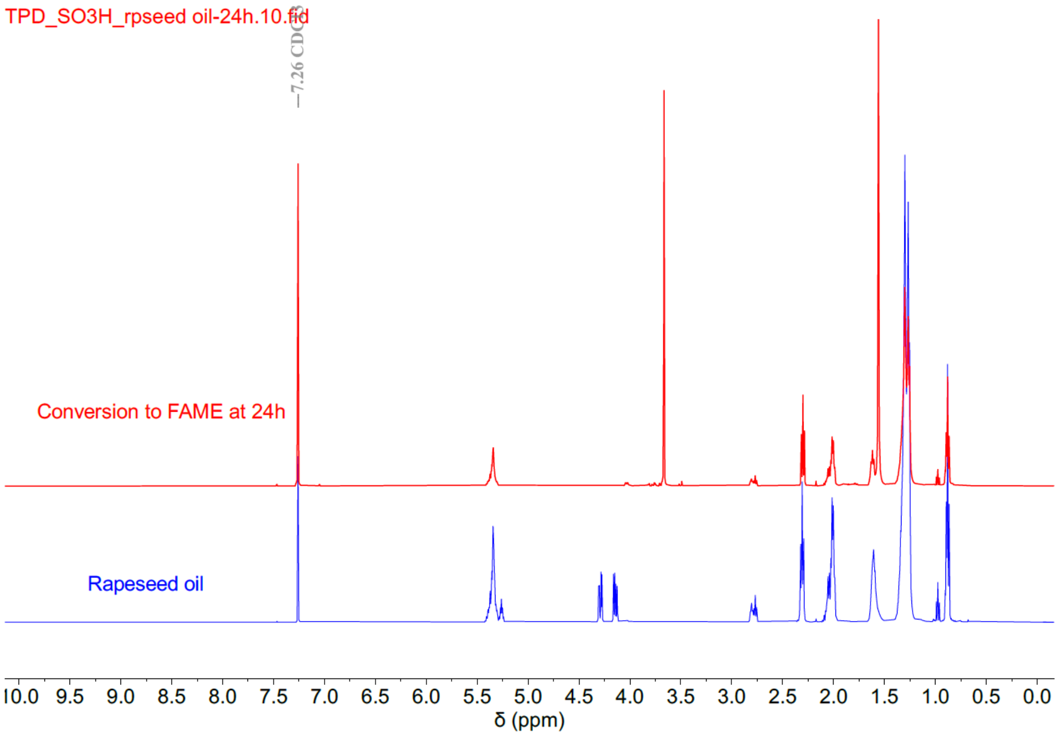

Figure S22: Conversion of rapeseed oil to the correspondent FAME with TPB-SO_3H

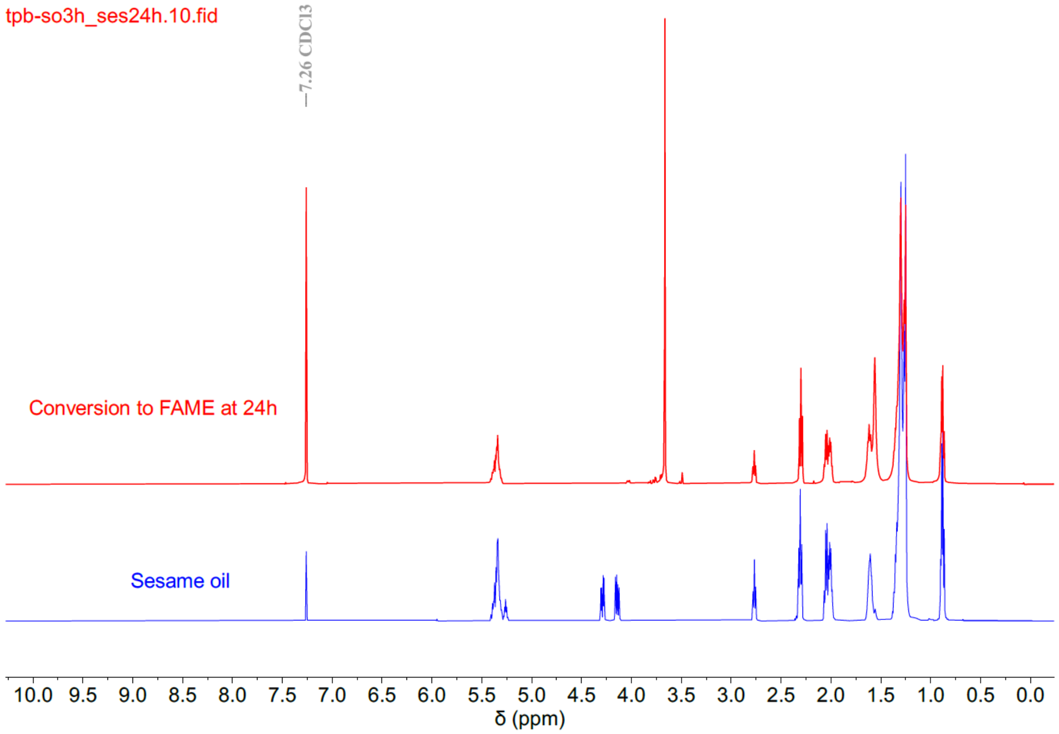

Figure S23: Conversion of sesame oil to the correspondent FAME with TPB-SO_3H

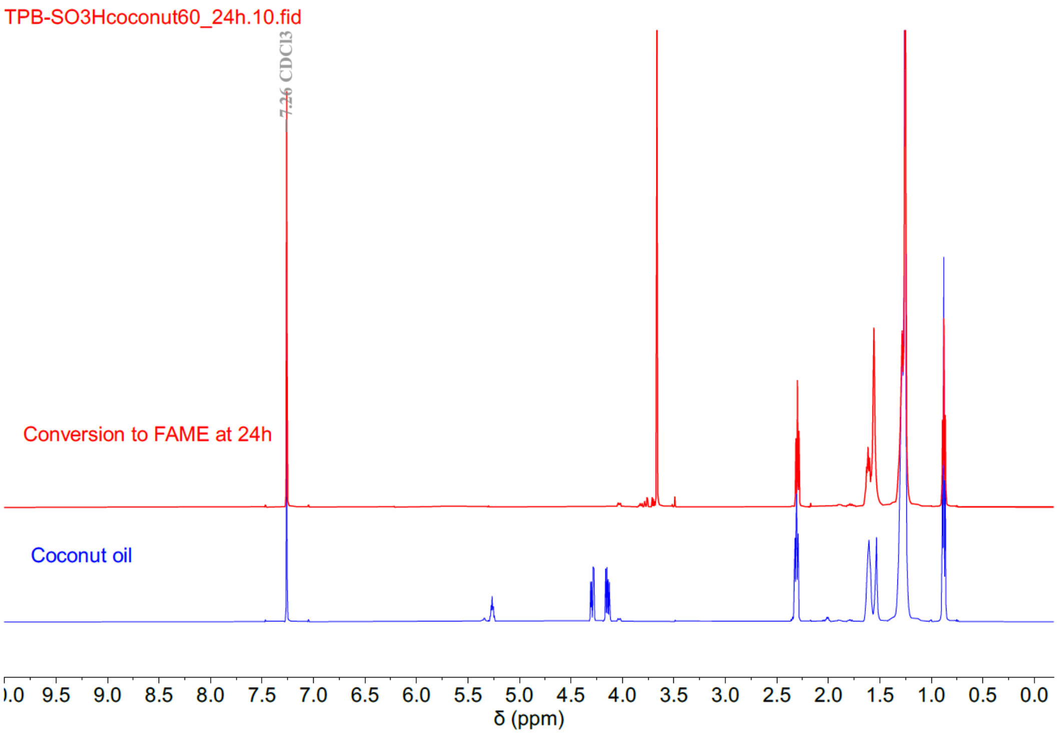

Figure S24: Conversion of coconut oil to the correspondent FAME with TPB-SO_3H

Table S1. Conversion of fatty acids into esters.

Palmitic Acid

Time (hours)	HPB-SO_3H	TPB-SO_3H	m-Ter-SO_3H
0	0	0	0
1	29	30	28
2	48	44	45
3	58	58	53
4	68	69	64
5	75	78	71
6	78	83	78
8	85	88	84
24	95	96	95

Lauric Acid

Time (hours)	HPB-SO_3H	TPB-SO_3H	m-Ter-SO_3H
0	0	0	0
1	30	26	23
2	46	42	50
3	58	62	63
4	69	71	70
5	75	78	76
6	79	83	80
8	85	88	84
24	96	95	95

Oleic Acid

Time (hours)	HPB-SO_3H	TPB-SO_3H	m-Ter-SO_3H
0	0	0	0
1	31	28	29
2	47	42	43
3	56	58	55
4	64	66	65
5	74	72	71
6	80	77	75
8	86	84	80
24	96	96	95

Stearic Acid

Time (hours)	HPB-SO_3H	TPB-SO_3H	m-Ter-SO_3H
0	0	0	0
1	25	30	21
2	40	44	39
3	52	58	51
4	61	66	60
5	68	73	66
6	74	78	72
8	83	84	80
24	96	96	95

Table S2. Conversion of triglycerides oils into esters.

Sesame Oil

Time (hours)	HPB-SO_3H	TPB-SO_3H	m-Ter-SO_3H
0	0	0	0
2	17	18	17
4	31	35	25
6	40	54	35
8	57	60	46
10	70	73	61
12	81	85	70
15	92	95	83
18	97	97	88
21	99	99	90
24	100	100	95

Coconut Oil

Time (hours)	HPB-SO_3H	TPB-SO_3H	m-Ter-SO_3H
0	0	0	0
2	15	16	11
4	35	34	22
6	47	52	32
8	55	62	40
10	70	68	52
12	80	75	60
15	92	88	66
18	97	93	74
21	100	100	81
24	100	100	89

Sunflower Oil

Time (hours)	HPB-SO_3H	TPB-SO_3H	m-Ter-SO_3H
0	0	0	0
2	21	24	13
4	40	37	23
6	53	58	34
8	69	68	44
10	77	75	56
12	87	85	66
15	94	90	80
17	95	93	86
20	96	95	93
24	99	98	95

Rapeseed Oil

Time (hours)	HPB-SO_3H	TPB-SO_3H	m-Ter-SO_3H
0	0	0	0
2	15	16	4
4	27	23	14
6	40	40	25
8	55	50	32
10	65	60	42
12	74	70	54
15	85	84	66
18	91	90	79
21	96	95	88
24	100	100	92

Composition of oils

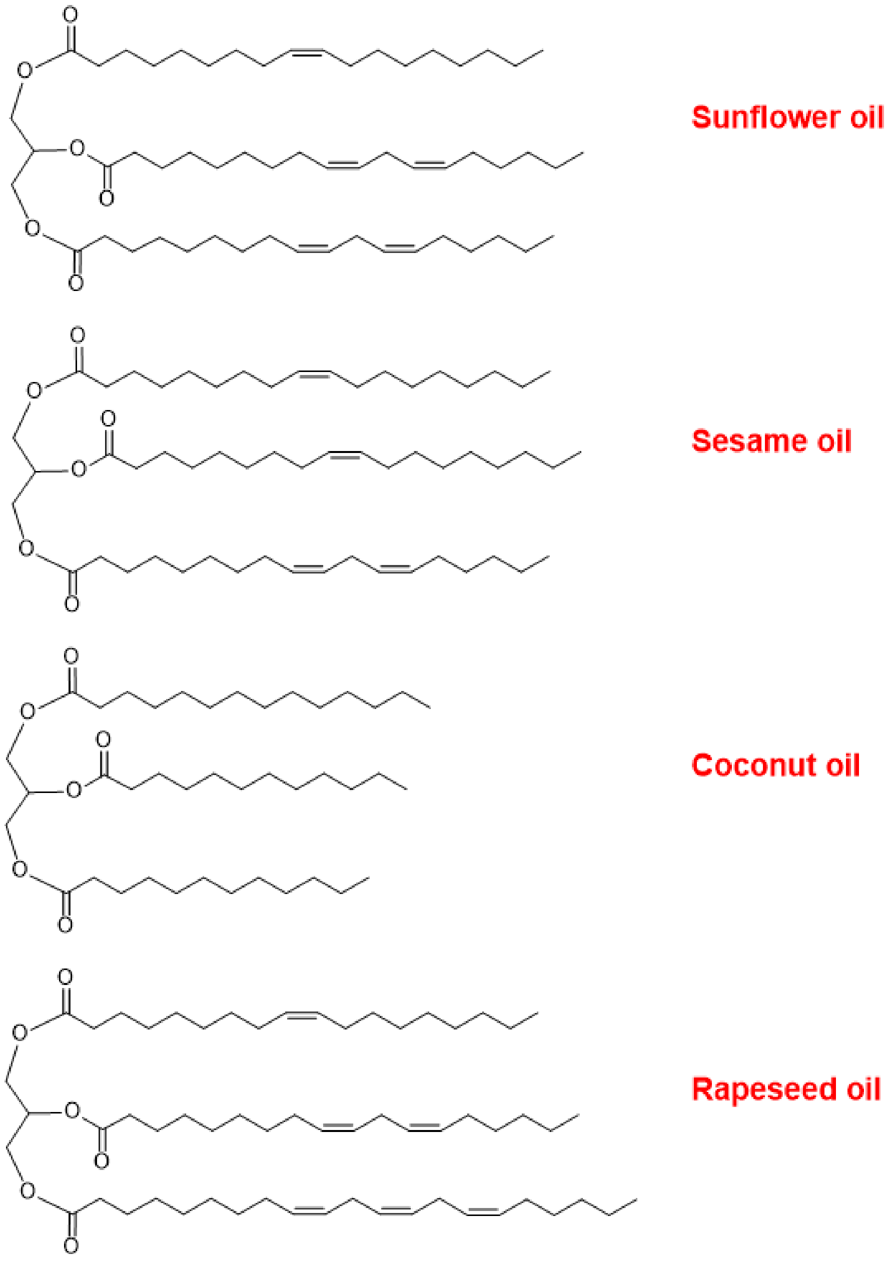

Figure S25. Structures of the different oils used for this work. The typical composition is:

Sunflower oil: Stearic acid (C18:0): ~6%; Oleic acid (C18:1): ~35%; Linoleic acid (C18:2): ~55% (+ minor others depending on variety). **Sesame oil**: Palmitic acid (C16:0): ~10%; Oleic acid (C18:1): ~30%; Linoleic acid (C18:2): ~55%. (+ minor others depending on variety). **Rapeseed:** Palmitic acid (C16:0): ~5%; Oleic acid (C18:1): ~60%; Linoleic acid (C18:2): ~30%. (+ minor others depending on variety). **Coconut**: Lauric (C12:0) ~45%; Myristic acid (C14:0): ~18%; Palmitic acid (C16:0) ~7%; Caprylic acid (C8:0): ~9%; Oleic acid (C18:1): ~5%; Linoleic acid (C18:2): ~1-2%. (+ minor others depending on variety)

^{13}C MAS SSNMR

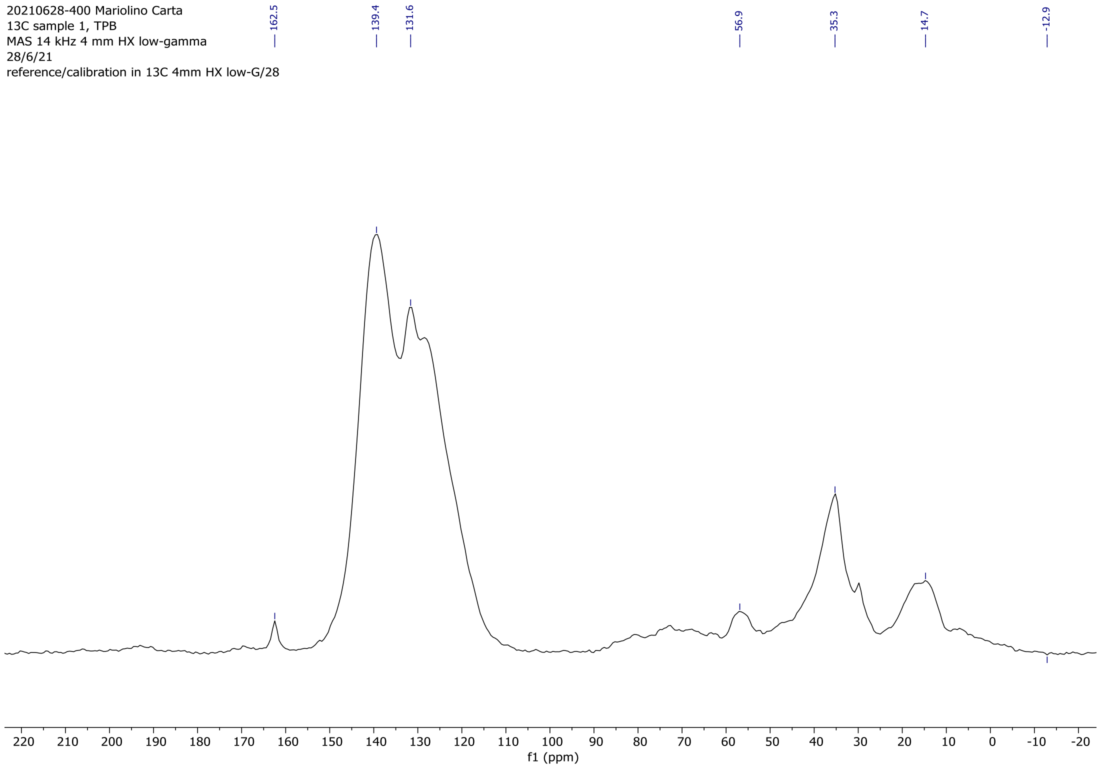

Figure S26. TPB-HC

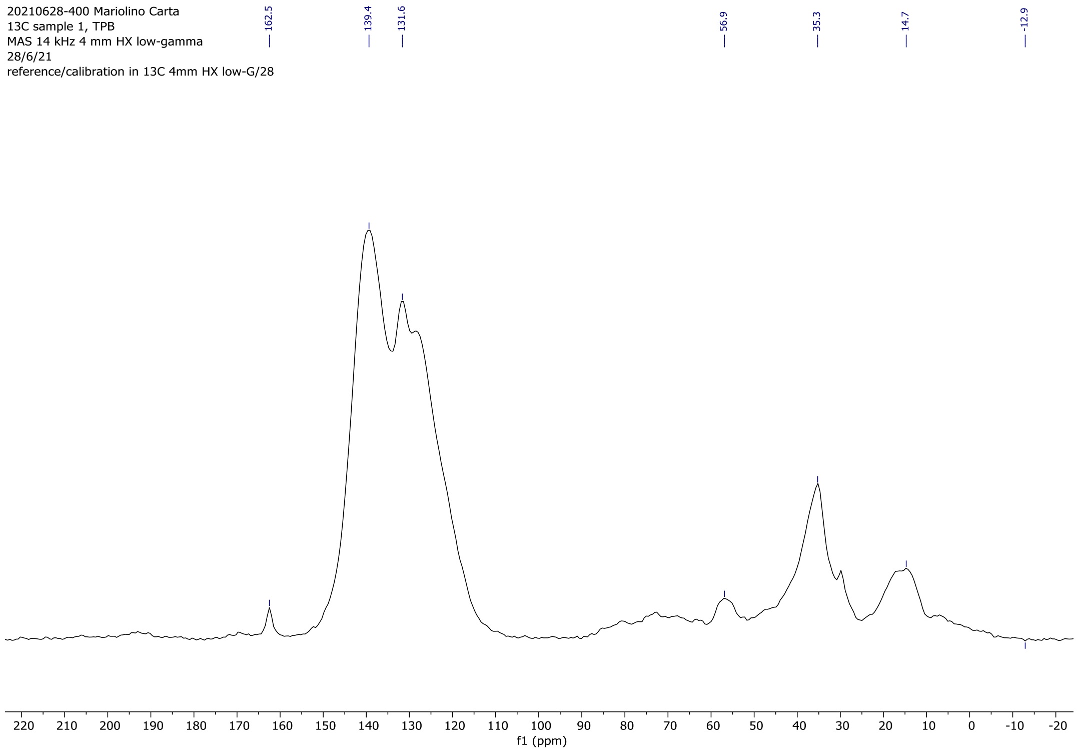

Figure S27. TPB-SO3H

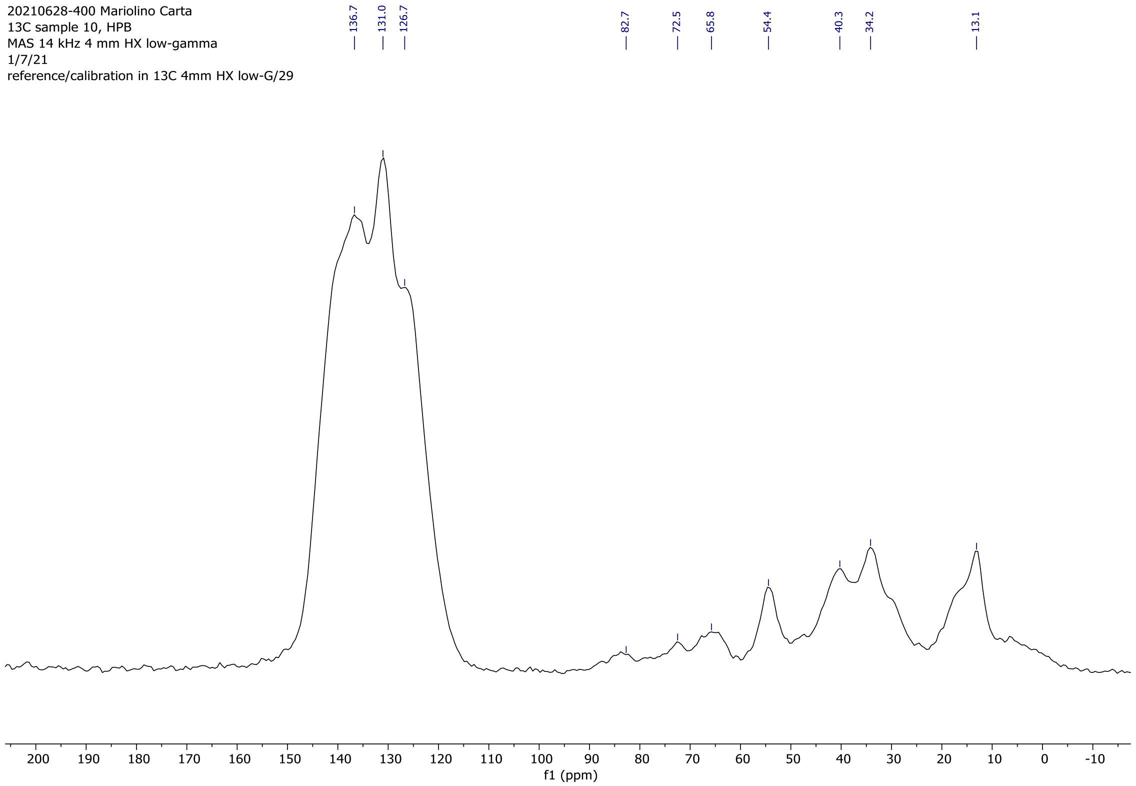

Figure S28. HPB-HC

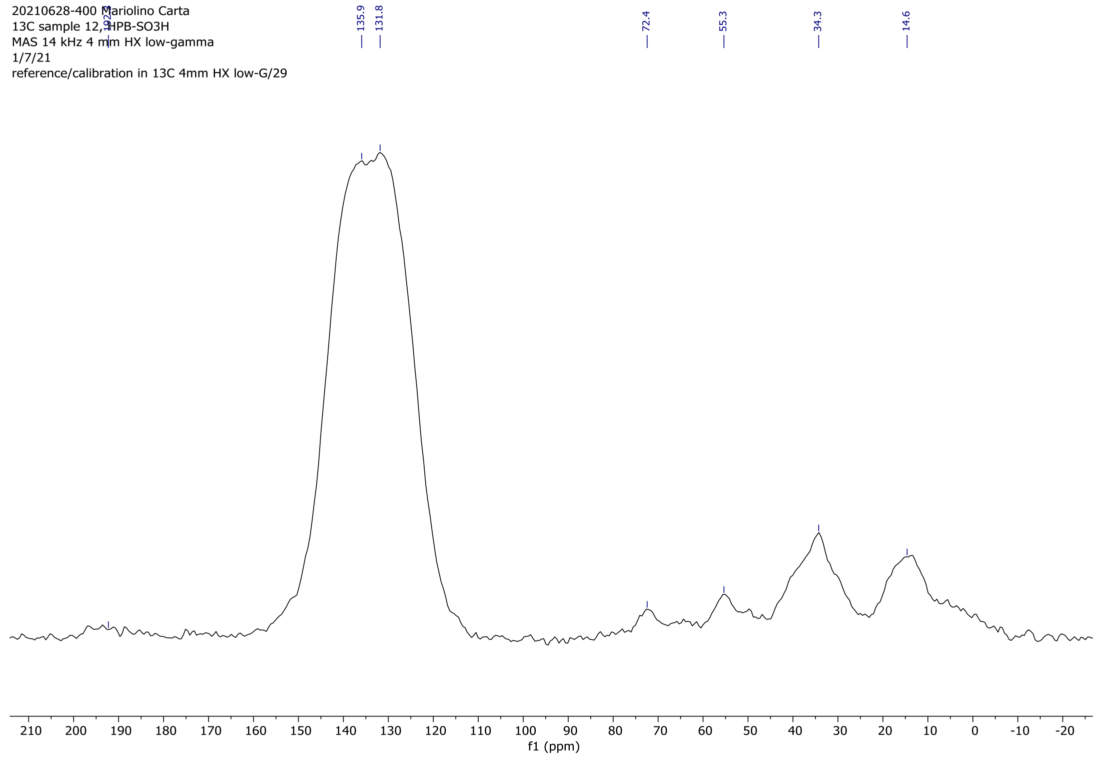

Figure S29. HPB-SO3H

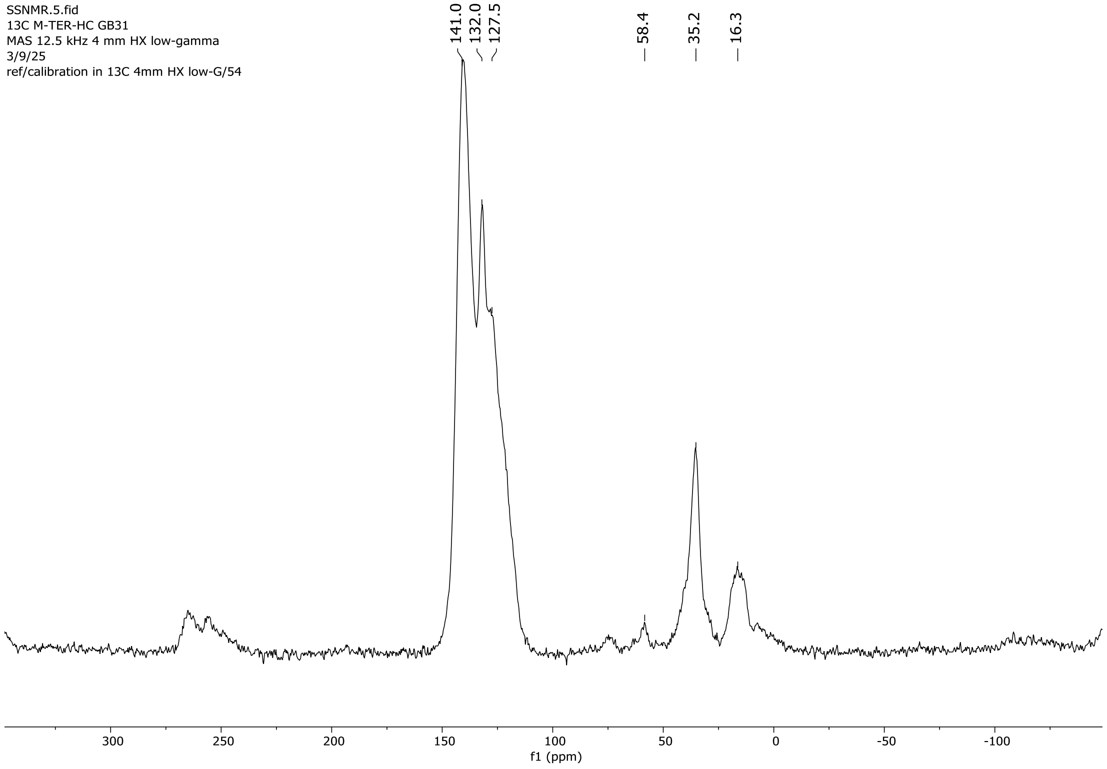

Figure S30. m-Ter-HC

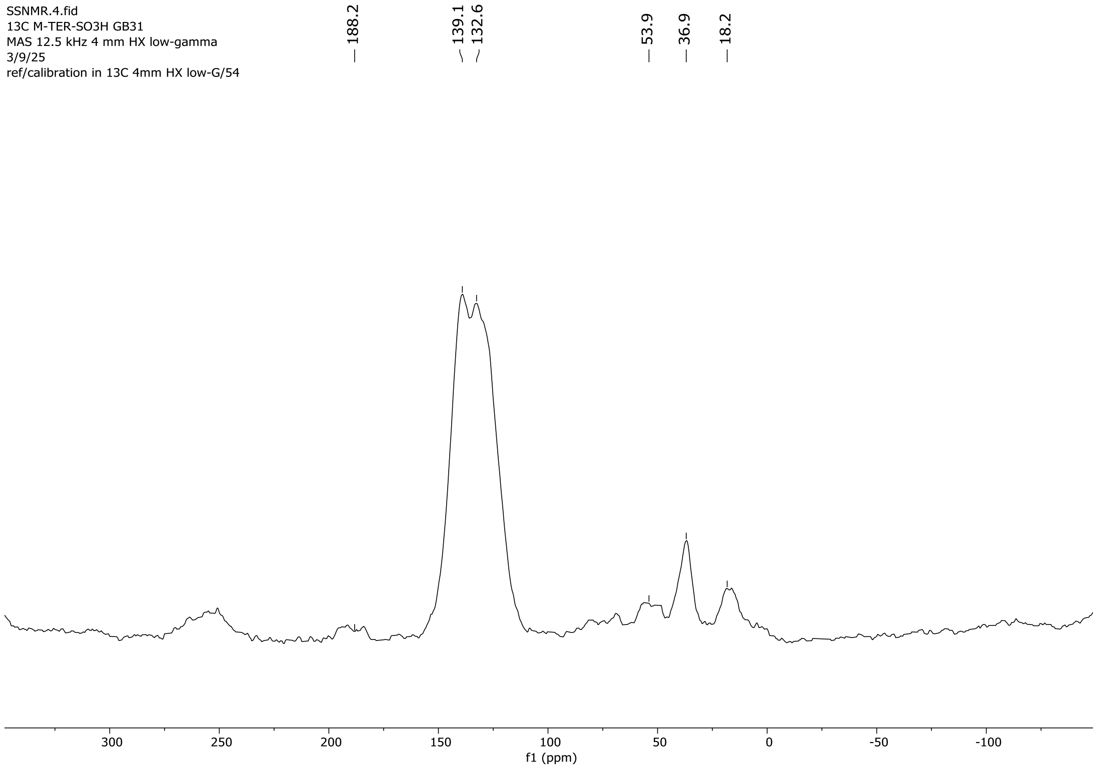

Figure S31. m-Ter-SO3H

SEM images

HPB-HC

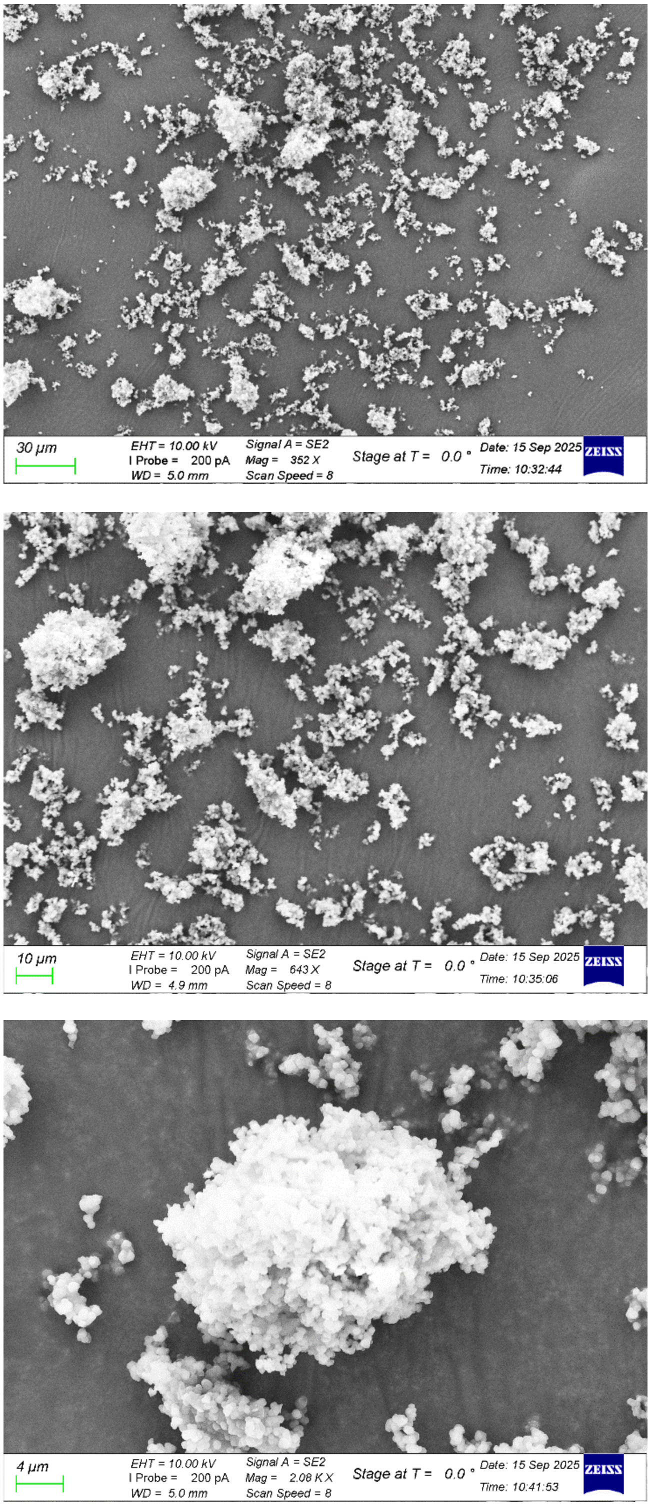

Figure S32. SEM of HPB-HC

HPB-SO_3H

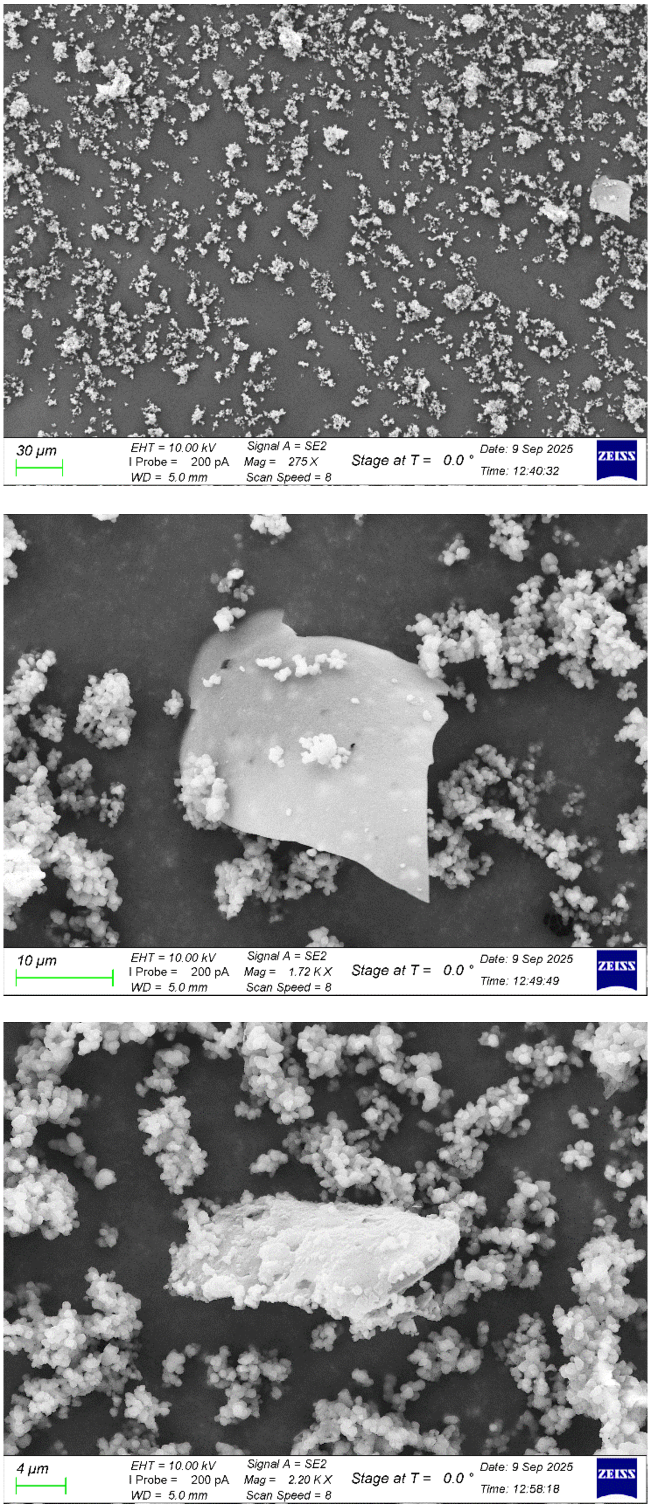

Figure S33. SEM of HPB-SO_3H

TPB-HC

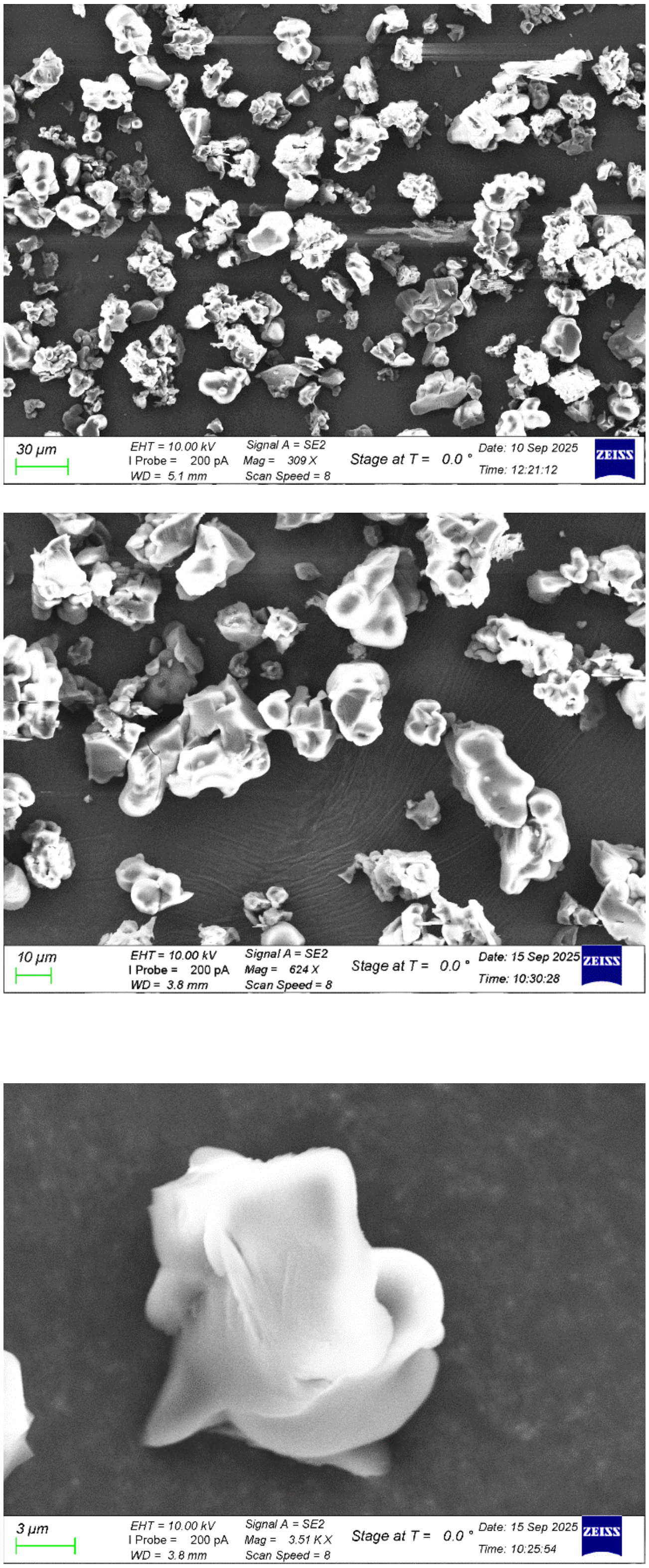

Figure S34. SEM of TPB-HC

TPB-SO_3H

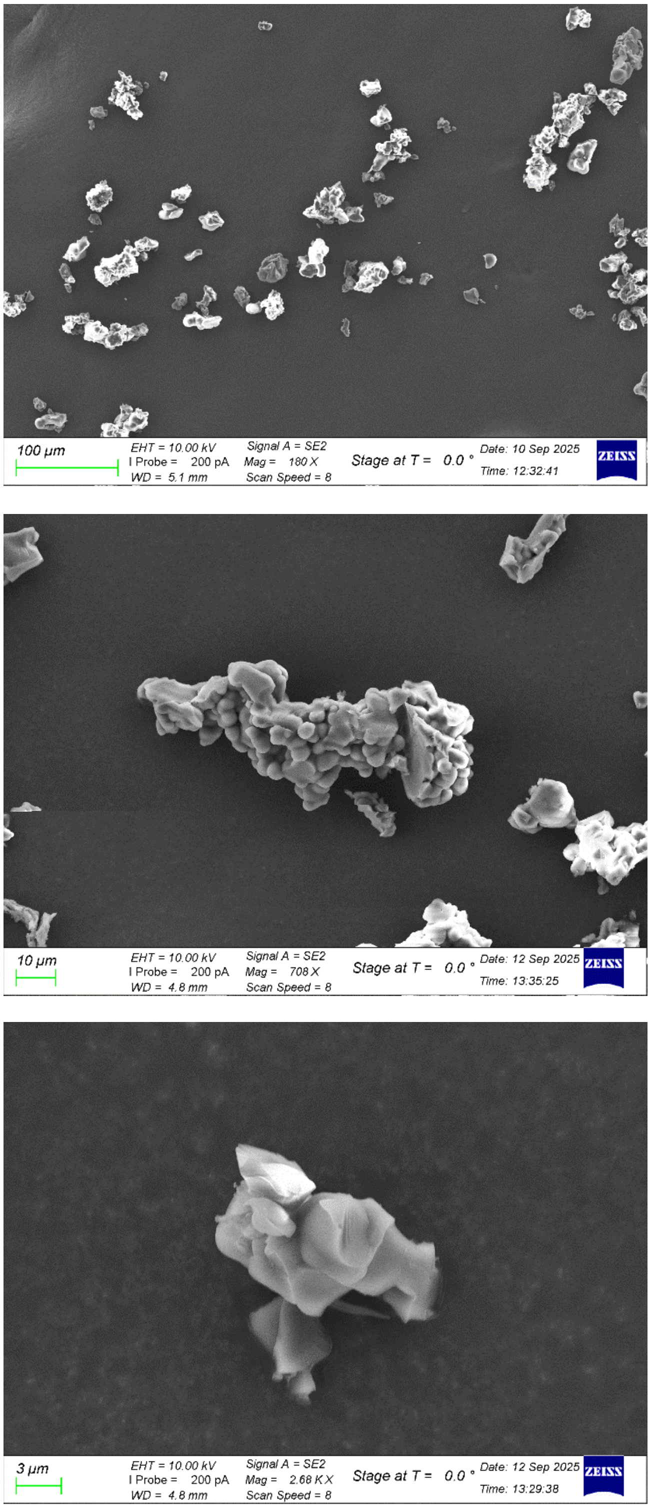

Figure S35. SEM of TPB-SO_3H

m-Ter-HC

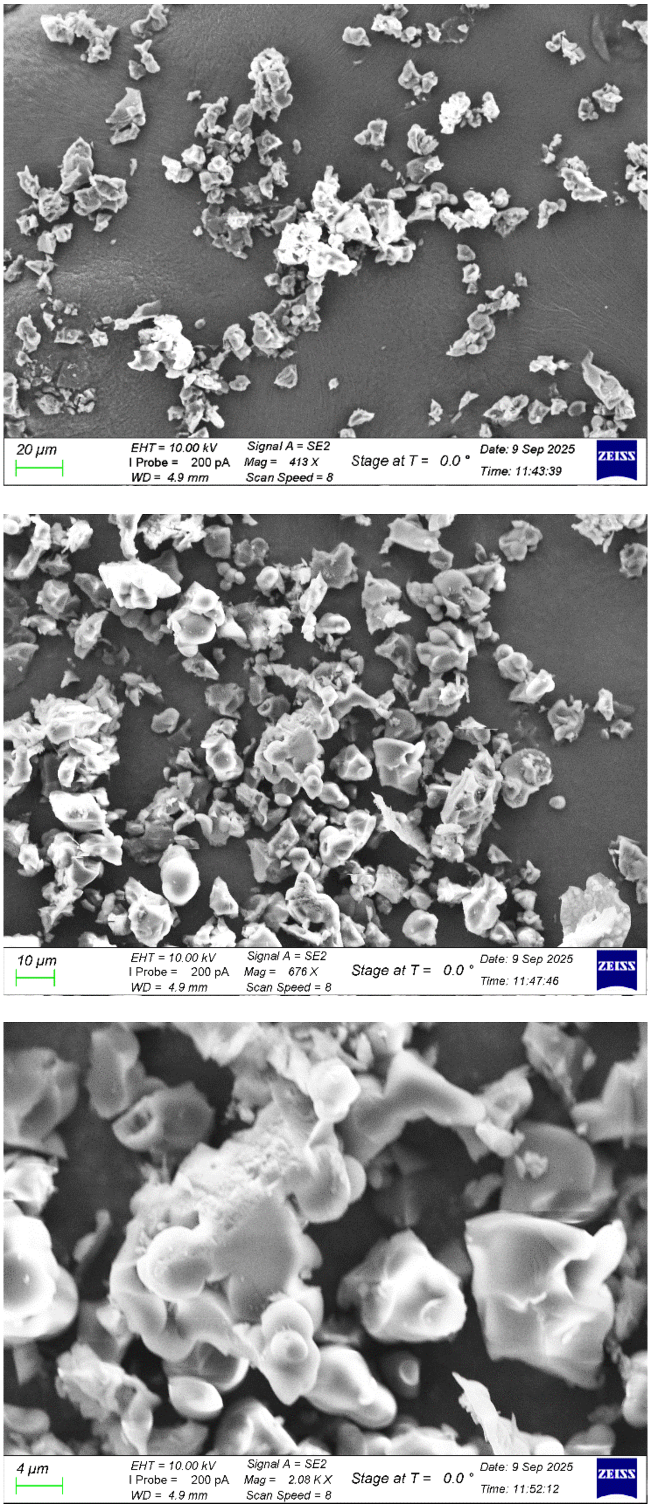

Figure S36. SEM of m-Ter-HC

m-Ter-SO_3H

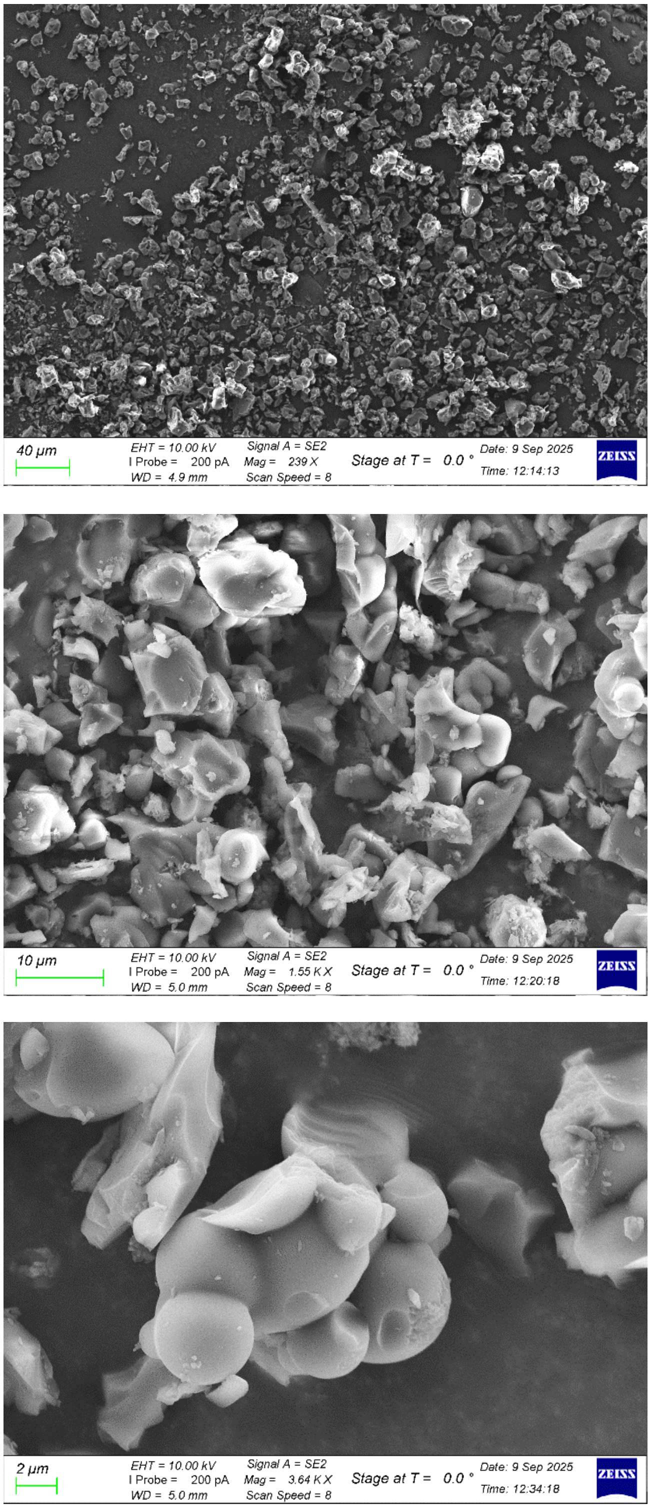

Figure S37. SEM of m-Ter-SO_3H

FT-IR of Polymers

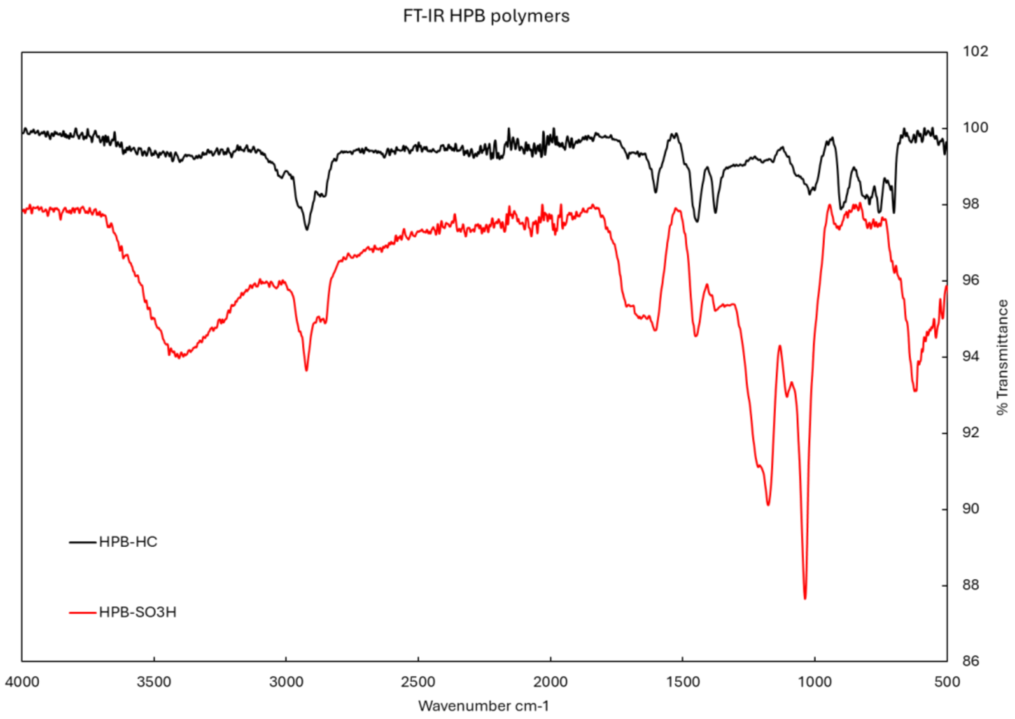

Figure S38. FT-IR of m-Ter Polymers

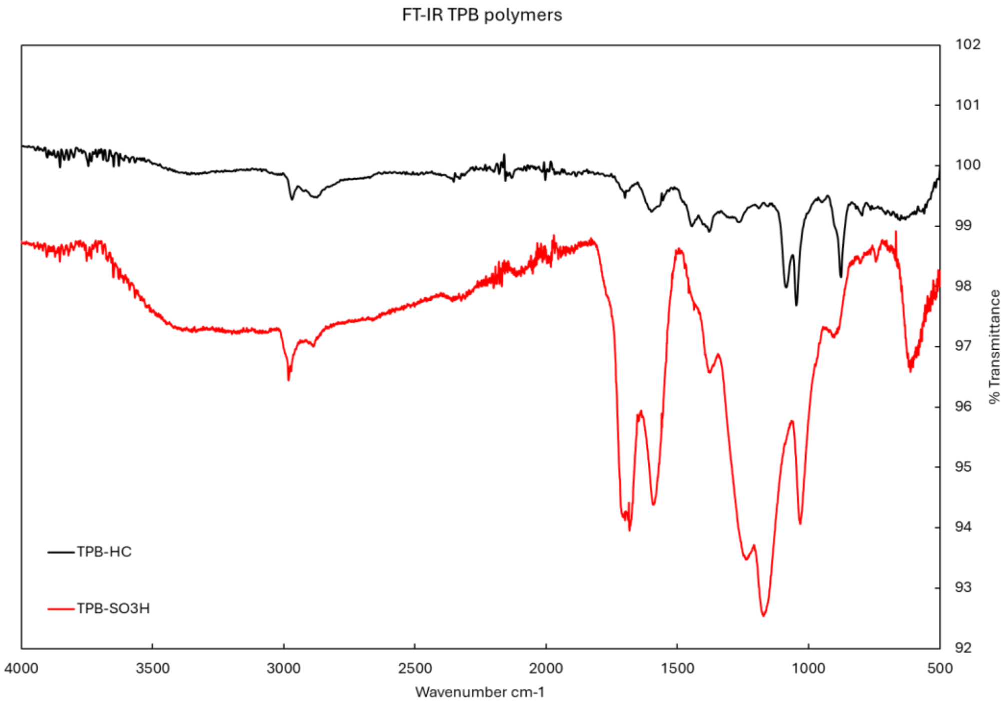

Figure S39. FT-IR of TPB Polymers

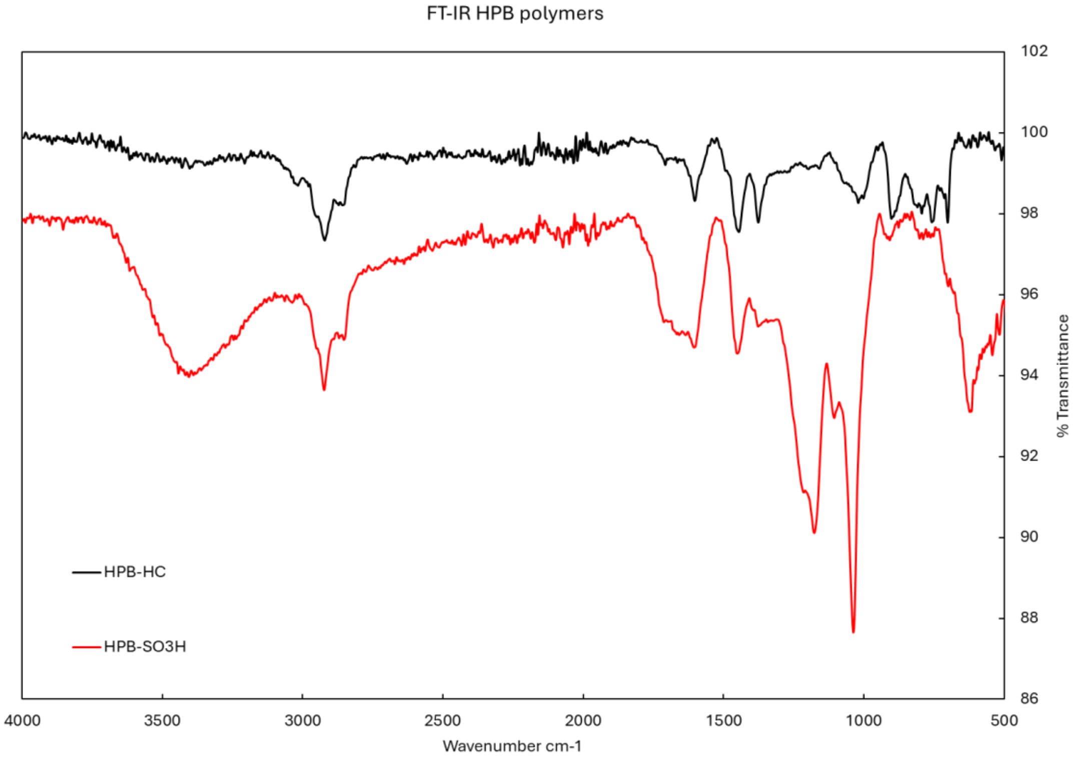

Figure S40. FT-IR of HPB Polymers

References

(1) Zhou, H.; Rayer, C.; Antonangelo, A. R.; Hawkins, N.; Carta, M. Adjustable Functionalization of Hyper-Cross-Linked Polymers of Intrinsic Microporosity for Enhanced CO2 Adsorption and Selectivity over N2 and CH4. *ACS Applied Materials & Interfaces* **2022**, *14* (18), 20997–21006.

(2) Al-Hetlani, E.; Amin, M. O.; Antonangelo, A. R.; Zhou, H.; Carta, M. Triptycene and triphenylbenzene-based polymers of intrinsic microporosity (PIMs) for the removal of pharmaceutical residues from wastewater. *Microporous Mesoporous Mater.* **2022**, *330*, 111602.

(3) Msayib, K. J.; McKeown, N. B. Inexpensive polyphenylene network polymers with enhanced microporosity. *J. Mater. Chem. A* **2016**, *4* (26), 10110–10113.
